# A Novel Prostate Cell Type-Specific Gene Signature to Interrogate Prostate Tumor Differentiation Status and Monitor Therapeutic Response (Running Title: Phenotypic Classification of Prostate Tumors)

**DOI:** 10.3390/cancers12010176

**Published:** 2020-01-10

**Authors:** Sarah N. Mapelli, Domenico Albino, Maurizia Mello-Grand, Dheeraj Shinde, Manuel Scimeca, Rita Bonfiglio, Elena Bonanno, Giovanna Chiorino, Ramon Garcia-Escudero, Carlo V. Catapano, Giuseppina M. Carbone

**Affiliations:** 1 Institute of Oncology Research (IOR), Università della Svizzera italiana (USI), 6500 Bellinzona, Switzerland; Sarah.mapelli@ior.usi.ch (S.N.M.); Domenico.albino@ior.usi.ch (D.A.); Dheeraj.shinde@ior.usi.ch (D.S.); 2 Swiss Institute of Bioinformatics (SIB), 1015 Lausanne, Switzerland; 3 Laboratory of Cancer Genomics, Fondazione Edo ed Elvo Tempia Valenta, 13900 Biella, Italy; maurizia.mellogrand@fondazionetempia.org (M.M.-G.); giovanna.chiorino@fondazionetempia.org (G.C.); 4 Department of Biomedicine and Prevention, University of Rome “Tor Vergata”, 00133 Rome, Italy; manuel.scimeca@torvergata.it (M.S.); Rita.bonfiglio@gmail.com (R.B.); Elena.bonanno@torvergata.it (E.B.); 5 Molecular Oncology Unit, CIEMAT, 28040 Madrid, Spain; 6 Biomedicine Research Institute, Hospital 12 octubre, 28040 Madrid, Spain; 7 Centro de Investigación Biomédica en Red de Cáncer (CIBERONC), 28040 Madrid, Spain; 8 Department of Oncology, Faculty of Biology and Medicine, University of Lausanne, 1011 Lausanne, Switzerland

**Keywords:** prostate cancer, tumor classification, predictive biomarkers, gene signature, gene classifier

## Abstract

In this study, we extracted prostate cell-specific gene sets (metagenes) to define the epithelial differentiation status of prostate cancers and, using a deconvolution-based strategy, interrogated thousands of primary and metastatic tumors in public gene profiling datasets. We identified a subgroup of primary prostate tumors with low luminal epithelial enrichment (LumE^low^). LumE^low^ tumors were associated with higher Gleason score and mutational burden, reduced relapse-free and overall survival, and were more likely to progress to castration-resistant prostate cancer (CRPC). Using discriminant function analysis, we generate a predictive 10-gene classifier for clinical implementation. This mini-classifier predicted with high accuracy the luminal status in both primary tumors and CRPCs. Immunohistochemistry for COL4A1, a low-luminal marker, sustained the association of attenuated luminal phenotype with metastatic disease. We found also an association of LumE score with tumor phenotype in genetically engineered mouse models (GEMMs) of prostate cancer. Notably, the metagene approach led to the discovery of drugs that could revert the low luminal status in prostate cell lines and mouse models. This study describes a novel tool to dissect the intrinsic heterogeneity of prostate tumors and provide predictive information on clinical outcome and treatment response in experimental and clinical samples.

## 1. Introduction

Prostate cancer is the most common cancer and a leading cause of cancer death in western countries [1]. Clinical evolution of prostate cancer is highly heterogeneous ranging from indolent to very aggressive tumors that rapidly progress to metastatic disease [2]. Whereas localized tumors are curable, a substantial fraction of patients with metastatic prostate cancer treated with androgen deprivation therapy (ADT) rapidly progress to lethal castration-resistant prostate cancer (CRPC). A critical issue is the identification of factors that distinguish indolent from aggressive tumors and, among primary and metastatic tumors, those that are prone to develop resistance to ADT and will likely benefit from alternative therapeutic strategies. Therefore, knowledge of the genetic, molecular, and biological characteristics of the tumors might allow the discovery and implementation of targeted therapeutic approaches tailored to the individual patients to prevent disease recurrence and avoid treatment resistance.

Genomic analyses of prostate tumors have provided information about molecular features associated with disease progression and resistance to current treatments [3,4]. Previous studies have proposed classifications of prostate tumors based on molecular markers, biological criteria, and gene expression patterns to identify tumors with different risk of progression, recurrence, and treatment resistance [5,6,7,8,9]. However, a classification of primary cancers that is strictly based on prostate epithelial cell features and interrogates the degree of prostate-specific differentiation is lacking.

The prostate gland is composed of a pseudostratified epithelium containing highly differentiated luminal secretory cells and a minor fraction of basal cells, which are less differentiated and may represent the normal stem/progenitor cells in the prostate [5,7,10]. Although the issue of cell origin of human prostate cancer is still controversial, experimental evidence in mouse models indicates that both luminal and basal cells can act as tumor progenitor cells and give rise to prostate adenocarcinomas [11,12,13]. Nevertheless, primary prostatic adenocarcinomas, the most frequent type of prostate tumors, have a predominantly luminal phenotype characterized by androgen receptor (AR) positivity and active AR signaling [14]. Interestingly, metastatic CRPCs emerging after ADT and next-generation AR pathway inhibitors (ARPIs) have increasingly non-luminal features, suggesting that subpopulations of non-luminal, less differentiated cells with basal/stem-like or neuroendocrine features might come into play during disease progression and emergence of resistance [15]. This phenotypic plasticity could be ascribed to the expansion of preexisting non-luminal progenitor/stem cells or dedifferentiation of luminal-type progenitor cells under the selective pressure of the treatment [16].

In this study, we developed a novel approach to probe the representation of different cell phenotypes and the divergence from the normal epithelial cell differentiation in prostate tumors starting from gene expression data of bulk tumor tissues. The method is based on epithelial cell-specific gene signatures (*metagenes*) and could be used for prognostic indications and therapeutic guidance in prostate cancer patients.

Analysis of gene expression data from tumors must account for the high cellular heterogeneity of bulk tissue samples containing a mixture of different cell types. Several reports have described methods based on deconvolution of transcriptomic data obtained from normal tissue into cell type-specific profiles [17,18]. By calculating the enrichment scores from these profiles, cell type-specific composition, such as stromal, immune, or cancer stem cell prevalence, can be inferred in heterogeneous tissues like solid tumors [18]. Using a conceptually similar approach, we analyzed gene expression data from about 2000 prostate tumor samples in multiple datasets. To reach our goal, we applied a novel strategy to dissect within prostate tumors, the differential enrichment of prostate cell-specific metagenes representative of the luminal and basal epithelial cell phenotype. We hypothesized that prostate tumors with increasingly aggressive features and less prone to respond to ADT would display a progressive divergence from the normal epithelial state and an imbalance of the expression of the epithelial cell-specific metagenes.

We found that primary tumors exhibited highly variable luminal and basal enrichment scores. Notably, tumors with attenuated luminal metagene enrichment displayed more aggressive traits than tumors exhibiting high luminal enrichment. Low luminal enrichment was consistently associated with high Gleason score, increased biochemical recurrence, and reduced overall survival. Furthermore, CRPCs had prevalently attenuated luminal metagene expression, suggesting that the low luminal phenotype might predispose to castration resistance. To facilitate the clinical application of this concept, we also developed using discriminant function analysis (DFA), an integrated 10-gene classifier that accurately subdivided both primary and metastatic prostate tumors based on the degree of luminal enrichment. Interestingly, a single gene in the mini-classifier marking low luminal tumors was associated with high Gleason score and was highly predictive of metastatic behavior in hormone-naïve prostate tumors. Application of the metagene scoring system to genetically modified mouse models (GEMMs) further revealed a consistent association between low luminal enrichment and aggressive phenotypes, such as invasive and metastatic behavior. The metagene approach applied to GEMMs and human cell lines provided also insights into drugs that could be used to revert tumors to a higher luminal status and enhance treatment response or prevent emergence of resistance.

This study describes a novel approach to stratify prostate tumors based on biological features intrinsic to the prostate epithelium and reflecting the persistence or loss of the differentiated epithelial cell phenotype. Due to its simplicity and efficiency, after further preclinical validation, this method may be suitable for clinical application to assess the degree of luminal differentiation in individual tumors and guide prognostic assessment and therapeutic decisions.

## 2. Results

### 2.1. Prostate Cell-Specific Metagenes Dissect Tumor Phenotypic Variability

We applied a prostate-specific metagene approach to assess the prevalence and degree of differentiation of luminal and basal epithelial cells in tissue samples from primary and metastatic prostate tumors. The overall experimental approach is outlined in Supplementary Figure S1. Our working hypothesis was that deviations from the predominant enrichment of luminal-type cell features in the transcriptomic profile might inform on the biological characteristics of the individual tumors and allow a sub-classification in tumor subtypes with different prognostic and therapeutic relevance. To this end, we derived lists of genes or metagenes representative of the main cell components of the normal prostatic tissue and applied them to bulk transcriptomic data of human prostate cancers.

**Figure 1:**
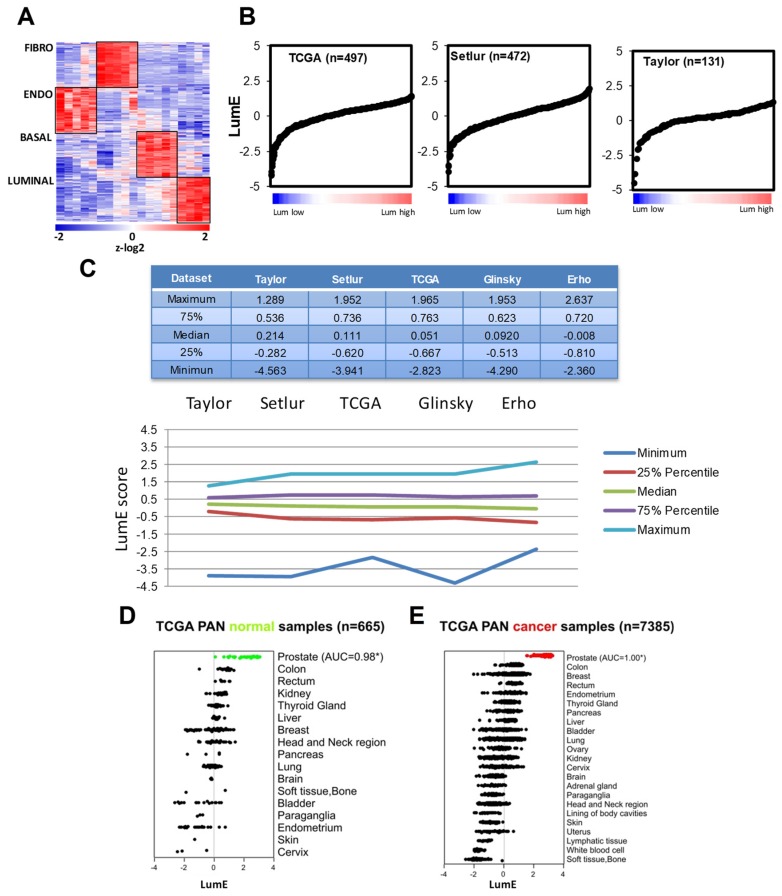
Extraction of prostate cell type-specific gene signatures and application to clinical prostate tumors. (**A**) Differential expression analysis was performed using *t*-test to extract genes selectively overexpressed among 4 different cell types of human prostate glands (see Section 4). Heatmap shows the unique expression patterns of each indicated cell type-specific metagene (**B**) Luminal metagene enrichment (LumE) scores using single-sample gene set enrichment analysis (GSEA) (ssGSEA) approach (see Section 4) in primary prostate cancer from the Taylor, Setlur, and The Cancer Genome Atlas (TCGA) datasets. Samples were ordered with increasing score values. (**C**) Key descriptive parameters of the distribution of luminal metagene scores are reported and plotted for the indicated datasets. NES, normalized enrichment scores. (**D**,**E**) LumE scores in normal (**D**) and cancer (**E**) tissue samples from TCGA-pancancer dataset. Samples are grouped by organ site and ordered from highest to lowest median value. Area under the receiver-operator characteristic (ROC) Curve (AUC) values are indicated. AUC values close to or equal to 1.00 (with asterisks) significantly classify prostatic versus non-prostatic tissue (normal or cancer tissue). Metagene enrichment scores are shown at the z-log_2_ scale.

To generate cell type-specific metagenes, we took advantage of published transcriptomic data of the main cell types in the human prostate gland: i.e., luminal, basal, stromal fibromuscular, and endothelial cells [10]. Transcriptomic profiling was performed on the four cell populations isolated by magnetic cell sorting (MACS) with cell type-specific monoclonal antibodies from benign tissue areas obtained from radical prostatectomy specimens [10]. The set of monoclonal antibodies applied for MACS included CD104 or ITGB4 for basal cells, CD26 or DPP4 for luminal secretory cells, CD49a or ITGA1 for stromal fibromuscular cells, and CD31 or PECAM1 for stromal endothelial cells. Gene expression data were generated using the Affymetrix platform (U133Plus 2.0 GeneChip). Applying differential gene expression analysis to the transcriptomic data, we extracted lists of genes that were highly differentially expressed between the four cell types and exhibited high cell type specificity (Figure 1A) (see Section 4). The gene lists or metagenes represented the transcriptome of the four cell types in the normal context and contained many molecular features already described for each specific cell type (Supplementary Table S1). Accordingly, basal keratins (KRT4, KRT5, KRT14, KRT15) were enriched in the basal metagene (BM). Mesenchymal transcription factors (TWIST1/2, ZEB2) were enriched in fibromuscular stroma metagene (FSM), while the endothelial stroma metagene (ESM) contained genes specific of endothelial cells. The luminal metagene (LM) was particularly enriched of genes highly expressed in the prostate gland (e.g., TRIM36 and members of the solute carrier family) and regulated by androgens [19].

Next, we applied a deconvolution strategy based on single-sample gene set enrichment analysis (ssGSEA) [8,20] using the generated metagenes to interrogate the transcriptome of human primary prostate tumors in a series of published datasets (Figure 1B and Supplementary Table S2). To overcome potential bias due to the different profiling platforms of the original studies, we used z-log transformation and standardization (see Section 4), which allowed direct comparison of the enrichment scores across the different datasets. Thus, for each sample, we calculated the enrichment score or single-sample metagene (SSM) score for the four metagenes. Inspecting the SSM scores for the luminal and basal metagenes across Taylor dataset [3], we observed variable degrees of enrichment of both the luminal and basal score (Supplementary Table S3). Notably, a discrete number of primary prostate tumors displayed very low luminal enrichment (LumE) compared to the majority of prostate cancer exhibiting prevalently high LumE score values (Figure 1B). A similar variance was observed for the enrichment of the basal metagene with a number of tumors exhibiting unexpectedly high basal enrichment (BasE) scores (Supplementary Figure S2A). We found similar variability in the LumE and BasE scores when we applied the metagenes to additional datasets (TCGA HiSeqV2_PANCAN_2014_08_22 release) [21], with a relevant fraction of tumors exhibiting a sharp decline in luminal metagene score (Figure 1B) and an increase in basal enrichment (Supplementary Figure S2A). SSM scores for endothelial (EndoE) and fibromuscular (FibroE) metagene showed a more limited variance across the tumor samples in all the datasets (Supplementary Figure S2B,C). Notably, comparing the metagene enrichment scores in multiple datasets, we observed similar distribution, median, and deviation from the median of the enrichment score values across all the datasets despite the use of different transcriptome profiling technologies (Supplementary Figures S3 and S4). In particular, the normalized enrichment scores (NES) for the luminal metagene were highly consistent across the different cohorts (Figure 1C). This supports the validity of our approach to interrogate prostate metagene enrichment using different technology platforms and data sources. Moreover, changes in LumE and BasE scores reflect increased divergence from the predominant luminal phenotype and dedifferentiation in a subset of primary tumors.

The luminal metagene was differentially enriched in normal prostate (*n* = 52) compared to other normal tissue samples (*n* = 613) including 17 different anatomical locations (Figure 1D). Furthermore, based on the area under the curve (AUC) of the receiver-operator characteristic (ROC), the luminal metagene displayed high ability to classify prostate versus non-prostate normal tissue samples (AUC = 0.98), indicating that the luminal metagene was very selective for the prostatic tissue despite an expected similarity with other epithelial tissues such as breast and bladder. Moreover, the luminal metagene score was significantly higher in prostate cancers and distinguished with very high accuracy (AUC = 1.00) prostate cancer from non-prostate cancer tumor samples (Figure 1E). The basal metagene was enriched also in normal prostate. However, other normal epithelial tissue (i.e., bladder, breast) had similar high values (Supplementary Figure S5). Furthermore, the basal metagene, despite a good performance in discriminating prostate versus non-prostatic normal tissue samples (AUC = 0.80), was unable to identify selectively prostate cancers among other tumor tissue samples (AUC = 0.52) displaying similar score distributions across many tumor types (Supplementary Figure S5). The fibromuscular metagene score was not significantly different between prostatic and non-prostatic tissues among both normal and tumor samples, whereas the endothelial metagene displayed the lowest scores in normal and cancer prostate samples (Supplementary Figure S5).

Collectively, these data indicated that the luminal metagene reflected core components of the transcriptome of normal prostate epithelial cells and accurately identified both normal and malignant prostatic tissues among other tissue types, making it a reliable metagene to monitor the epithelial cell differentiation state in normal and tumor prostatic tissue samples. Interestingly, the evaluation of the basal, fibromuscular, and endothelial metagenes in the subgroup of tumors with low LumE compared to non-low LumE tumors revealed that those with low luminal enrichment displayed unusual high BasE, EndoE, and FibroE scores (Supplementary Figure S6A), suggesting that loss of luminal characteristics was associated with epithelial dedifferentiation and changes in cellularity.

### 2.2. Low Luminal Tumors Exhibit Poor Clinical Outcome and Increased Mutational Burden

**Figure 2:**
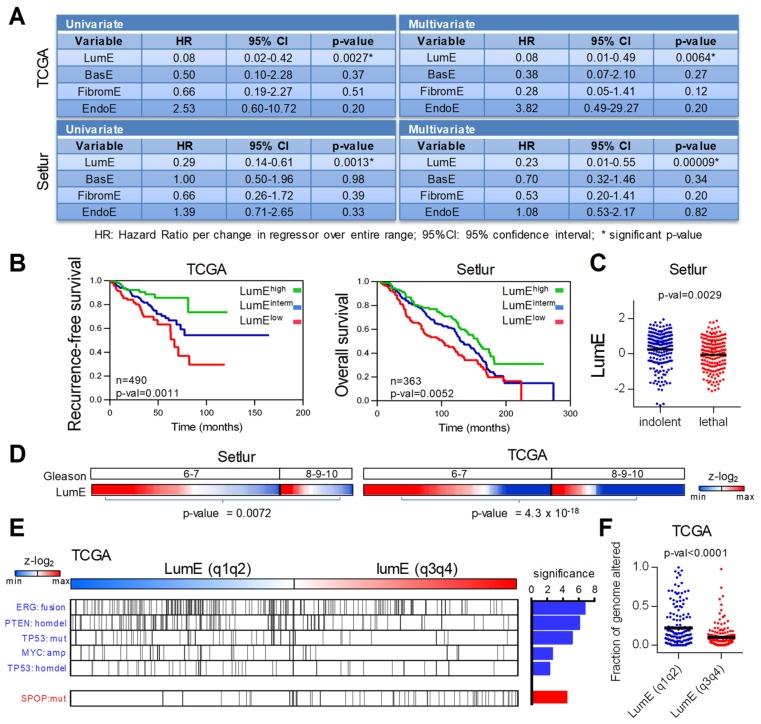
Luminal metagene is associated with aggressive features and poor prognosis. (**A**) Univariate and multivariate Cox regression analysis in the TCGA and Setlur datasets (recurrence-free survival and overall survival, respectively) using LumE, BasE, FibromE, and EndoE metagene scores. LumE is significantly associated with poor prognosis in both cohorts. Association with other metagenes was not significant. (**B**) Kaplan–Meyer analysis of recurrence-free survival (TCGA) and overall survival (Setlur) based on LumE score. Patients were allocated in 3 groups depending on score values: LumE^low^ (quartile q1), LumE^intermed^ (quartiles q2 and q3), and LumE^high^ (quartile q4). (**C**) Comparison of LumE scores in indolent and lethal prostate tumors in the Setlur dataset. P-value calculated with *t*-test. (**D**) Correlation between LumE and Gleason scores in Setlur and TCGA datasets. High Gleason samples display significantly lower LumE scores in both cohorts. P-values calculated with *t*-test. (**E**) TCGA samples with lower LumE scores (quartiles q1 and q2) display more genetic alterations than those having higher scores (quartiles q2 and q4). Significant differences were calculated with Fisher’s exact test (threshold *p*-value < 0.01). Significance: −log_10_ (*p*-value). Blue bars: more alterations in LumE q1q2. Red bar: more alterations in LumE q3q4. (**F**) Association between luminal score and fraction of genome altered in TCGA dataset. Samples with lower LumE scores (q1q2) display higher fraction of genome altered than higher LumE scores (q3q4). Metagene enrichment scores are shown at the z-log_2_ scale.

The luminal metagene appeared as a reliable tool to monitor the epithelial differentiation state in prostate tumors. To determine whether the luminal metagene was associated with clinical outcome, we performed Cox regression analysis overall survival and biochemical recurrence (Figure 2A). Univariate and multivariate Cox regression analysis showed a significant association of the LumE score with adverse prognosis for both overall and recurrence-free survival. Conversely, no associations were seen with the BasE, FibromE, and EndoE scores. Kaplan–Meyer analysis for recurrence-free survival and overall survival demonstrated that patients with low LumE tumors displayed poorer outcome than those with high and intermediate LumE score (Figure 2B). We used also an immune signature generated in an independent study to detect and quantify the level of immune infiltrates from transcriptomic data in complex tissue samples [18]. The immune signature score did not show any significant association with survival in the Taylor and Setlur cohorts of primary prostate tumors (Supplementary Figure S6B). Interestingly, low luminal tumors exhibited on average higher immune signature enrichment scores than non-LumE low tumors, as also seen with the other metagenes (Supplementary Figure S6C). Furthermore, LumE scores were significantly lower in lethal prostate cancers than indolent tumors (Figure 2C). Primary prostate cancer with higher (≥8) Gleason score had also significantly lower LumE (Figure 2D). Thus, low LumE score was predictive of clinically aggressive disease, whereas none of the other metagenes *per se* had an impact on clinical outcome.

Notably, using genomic data available for tumors in the The Cancer Genome Atlas (TCGA) dataset we found a higher frequency of genetic alterations in low LumE (q1q2) tumors. Specifically, tumors with low luminal enrichment displayed significantly higher frequencies of ERG gene fusion, PTEN homozygous deletion, TP53 homozygous deletion and mutation, and MYC amplification (Figure 2E). In contrast, SPOP mutations, which are generally associated with better prognosis [22], were more frequent in tumors with high LumE (q3q4). The overall mutational burden was also significantly higher in low LumE (q1q2) tumors compared to high LumE (q3q4) tumors (Figure 2F). The higher frequency of mutations corroborates our hypothesis that the level of luminal enrichment may reflect the phenotypic plasticity associated with disease progression, independently of the nature of the specific mutations identified in advanced tumors.

### 2.3. Association of Prostate-Specific Features in LumE-Defined Tumor Subtypes

**Figure 3:**
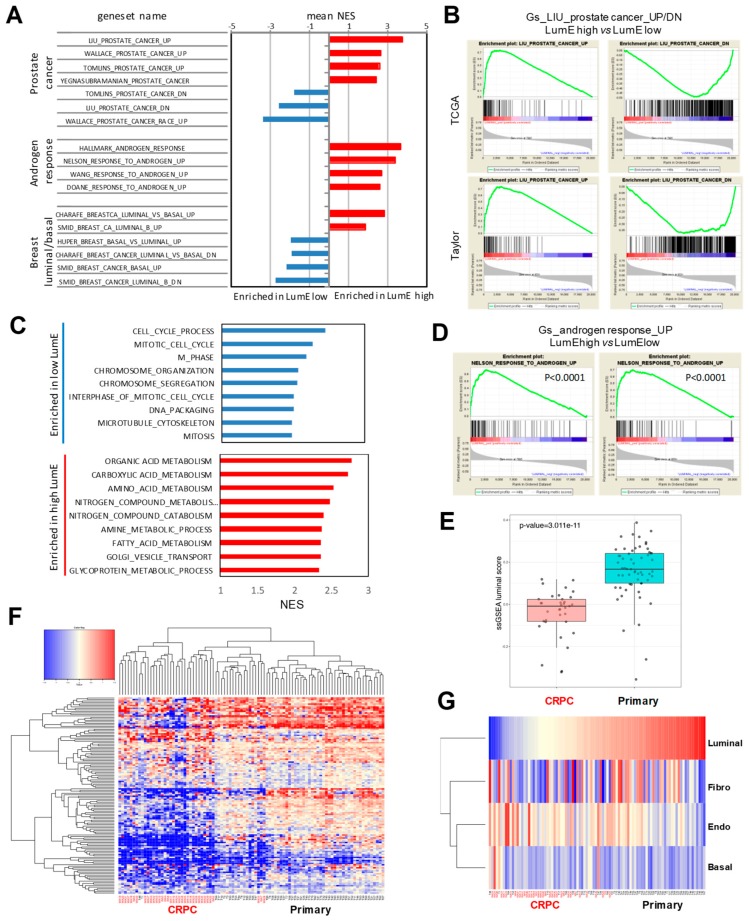
Luminal metagene score is associated with androgen receptor signaling and is attenuated in castration-resistant prostate tumors. (**A**) Gene set enrichment analysis (GSEA) in tumors with higher or lower LumE scores in The Cancer Genome Atlas (TCGA) and Taylor datasets. NES, normalized enriched scores. (**B**) GSEA plots with prostate-specific genesets showing enrichment in tumors with high LumE scores. (**C**) GSEA with KEGG pathways showing enrichment in tumors in TCGA dataset with either higher or lower LumE scores. (**D**) GSEA plots with androgen upregulated genes showing enrichment in high LumE tumors in Taylor (left) and TCGA (right) datasets. (**E**) Luminal scores in primary tumors versus castration-resistant prostate cancers (CRPCs) in the Grasso dataset. Luminal scores are significantly lower in CRPCs. (**F**) Unsupervised hierarchical clustering analysis of primary tumors and CRPCs based on the luminal metagene. Note the robust clusterization of CRPCs based on the attenuated luminal signature. (**G**) Distribution of metagene scores in primary tumors and CRPCs. Samples are ordered by increasing LumE scores. Note the prevalently lower LumE scores in CRPCs compared to primary tumors. For GSEA threshold of significance is FDR < 0.01.

We applied GSEA to identify molecular pathways differentially enriched in tumors with high and low LumE score in the TCGA and Taylor datasets. Primary tumors with high LumE profile were enriched in genes canonically upregulated in prostate cancer (Figure 3A,B; Supplementary Tables S4 and S5). Conversely, low LumE tumors were enriched of genes downregulated in prostate cancer (Figure 3A). We observed also a relationship between LumE score with genes either up or downregulated in luminal or basal breast cancers (BC) (Figure 3A). Thus, the luminal metagene enrichment reflected the degree of differentiation or loss of prostate epithelial markers. Interestingly, also tumors with a high BasE profile exhibited features canonically downregulated in prostate cancers, whereas the opposite occurred in low BasE tumors (Supplementary Figure S7). Notably, functional annotation analysis revealed enrichment of metabolic and secretory pathways in tumors with high LumE score, whereas molecular pathways related to proliferation, mitogenesis, and chromatin dynamics were positively associated with low LumE tumors (Figure 3C).

Responsiveness to androgen stimulation is an important aspect of normal luminal epithelial cells. Thus, the degree of luminal enrichment could be a surrogate marker of AR signaling proficiency and sensitivity to AR-targeting therapy. Interestingly, application of the metagene scoring system to gene expression data from LNCaP cells showed a significant time-dependent increase of the LumE score and a concomitant decrease of the BasE score upon androgen stimulation (Supplementary Figure S8A). Furthermore, androgen-activated genes were significantly enriched in tumors with high LumE score (Figure 3D), whereas genes downregulated by androgens were enriched in tumors with high BasE score (Supplementary Figure S8B). To examine further the relation between luminal metagene enrichment and susceptibility to AR signaling inhibition, we compared hormone-naïve primary tumors and CRPCs in distinct datasets [23,24]. Notably, both primary CRPCs and metastatic CRPCs had significantly lower LumE scores compared to hormone-naïve primary tumors, suggesting that decreased luminal differentiation may favor castration-resistance (Supplementary Figure S8C,D).

To further support the link between luminal metagene and castration resistance, we evaluated the metagene scores in primary tumors and CRPCs in a large dataset [25]. Consistent with our hypothesis, LumE scores were significantly lower among CRPCs than primary tumors (Figure 3E). Among the other metagenes, BasE and EndoE scores were not significantly different, whereas the average fibromuscular metagene score was significantly lower in CRPCs (Supplementary Figure S9). Strikingly, unsupervised hierarchical clustering showed that the luminal metagene separated primary tumors from CRPCs (Figure 3F) and, in supervised analyses, low LumE scores were preferentially associate with CRPCs (Figure 3G). With the exception of the FibroE, the other metagenes performed poorly in supervised analyses, being unable to discriminate between the two groups (Supplementary Figure S10).

**Figure 4:**
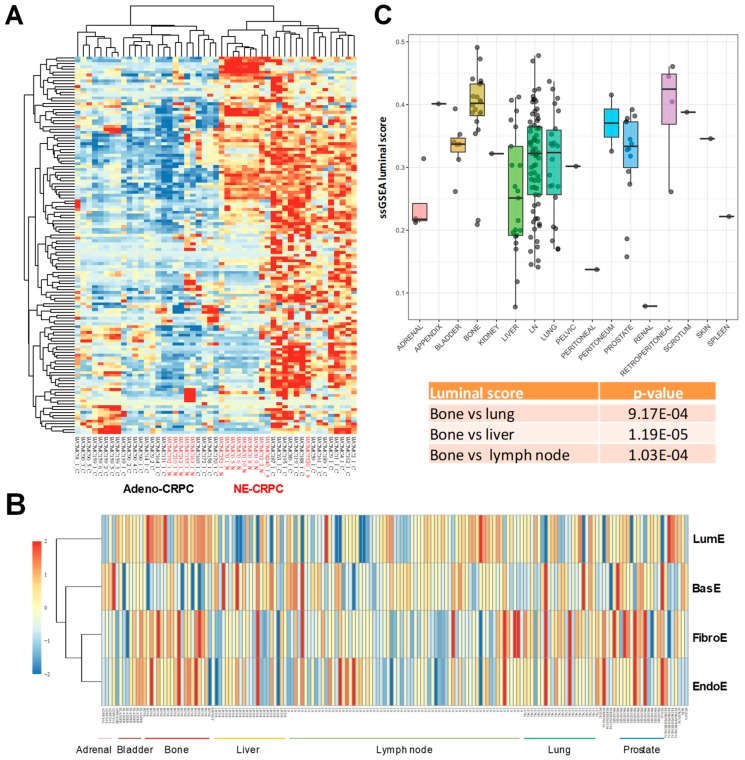
Enrichment scores in aggressive prostate tumors and metastases. (**A**) Unsupervised hierarchical clustering analysis of adenocarcinoma (Adeno) and neuroendocrine (NE) castration-resistant prostate cancers (CRPCs) based on luminal metagene expression. Note the presence of sub-clusters of both Adeno and NE CRPCs with different degrees of luminal enrichment. (**B**) Metagene score distribution in tissue samples from different metastatic sites. (**C**) Luminal enrichment scores in metastatic prostate tumors at distinct organ sites. *p*-values relative to the comparison of luminal enrichment scores between bone versus lung, liver, and lymph node metastases are indicated.

Increasing numbers of CRPCs, particularly after treatment with next-generation ARPIs, exhibit a neuroendocrine phenotype, a phenomenon that has been linked to phenotypic plasticity and dedifferentiation driving treatment resistance [16,26]. Applying the metagene approach we found independent clustering of neuroendocrine CRPCs (NE-CRPCs) compared to adeno-CRPCs (Figure 4A). Indeed, most NE-CRPCs formed in an intermediate heterogeneous group with adeno-CRPCs forming two distinct clusters with relatively higher and lower luminal enrichment, respectively. Indeed, sub-clusters of both NE and Adeno-CRPCs can be seen examining the clustering tree, which might reflect the evolution and transition of castration-resistant tumors through intermediate stages of dedifferentiation and loss of luminal phenotype during disease progression. Whether these sub-clusters of NE and Adeno-CRPCs with different retention of luminal features are associated with distinctive clinical features remains to be investigated.

Our data indicate that loss of epithelial cell differentiation is more evident in metastatic tumors. However, whether the luminal phenotype is retained at the metastatic sites is unknown. Using transcriptomic data from metastases of prostate cancer at distinct organ sites [27], we assessed the degree of retention or loss of luminal differentiation. We focused on the luminal metagene because of its association with clinical outcome in patients with primary tumors. Intriguingly, the level of luminal enrichment varied considerably among the distinct metastatic sites as seen by supervised clustering analysis (Figure 4B). Bone metastases had higher LumE scores, whereas lymph node and visceral (e.g., liver, lung) metastases exhibited mainly lower LumE scores (Figure 4C). The differences in LumE scores between bone and the other metastatic sites (i.e., lymph node, liver, and lung) were highly statistically significant. The other metagene scores also varied among metastatic sites, although the overall variance was reduced and only in few cases the difference between bone and visceral metastases was significant (Supplementary Figure S11). Thus, metastatic prostate cancer cells to the bone retain luminal features and likely AR signaling proficiency, which may make them relatively more responsive to AR modulators compared to visceral metastases. These data are consistent with the notion that AR is still expressed and active in these metastatic tumors as shown by Kumar et al. [27]. However, our study highlights for the first time a relative enrichment of luminal genes in bone versus visceral metastasis. This finding deserves further investigation. Collectively, these data indicate that the luminal metagene can identify, among both primary and metastatic tumors, those tumors that deviate from the canonical prostate-specific luminal-type profile and are more likely to progress to castration-resistance, metastatic, and lethal cancers.

### 2.4. Extraction of a Mini-Classifier Gene Set and Orthogonal Validation by Immunohistochemistry

**Figure 5:**
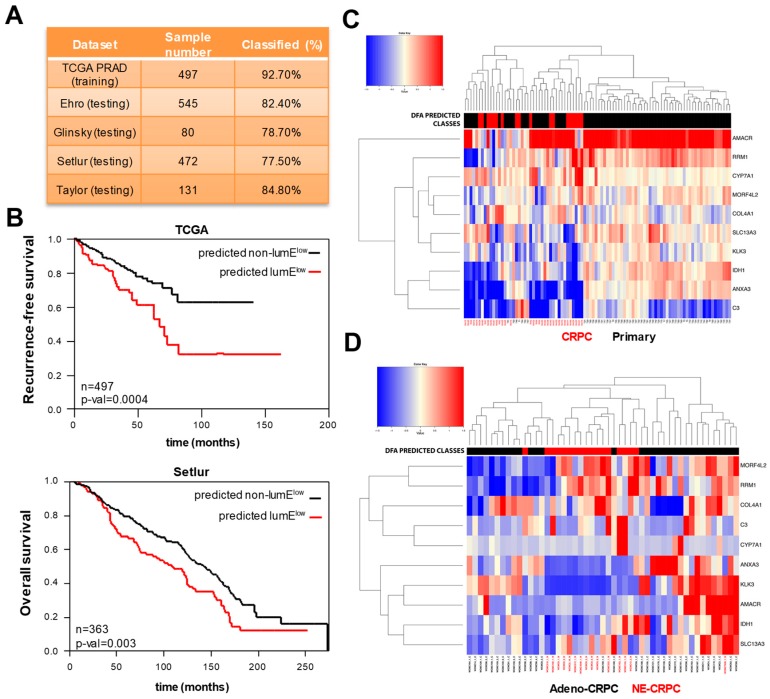
Extraction and validation of a 10-gene mini-classifier of prostate epithelial differentiation. (**A**) Percentage of correctly classified samples using the 10-gene classifier in prostate cancer datasets. The Cancer Genome Atlas (TCGA) cohort (training set) was used to obtain the predictor model using discriminant function analysis (DFA) and thereafter tested in Erho, Glinsky, Setlur, and Taylor (testing sets). (**B**) Kaplan–Meyer analysis of recurrence-free survival (TCGA) and overall survival (Setlur) based on the groups predicted by the 10-gene classifier. (**C**) Unsupervised hierarchical clustering of primary tumors and metastatic castration-resistant prostate cancers (CRPCs) based on the 10-gene classifier. Discriminant function analysis (DFA) predicted classes of low (red) and non-low (black) LumE tumors are indicated at the top. (**D**) Unsupervised hierarchical clustering of adenocarcinoma (adeno-CRPC) and neuroendocrine (NE-CRPC) CRPCs based on the 10-gene classifier. DFA predicted classes of low (red) and non-low (black) LumE tumors.

To facilitate the clinical application of the metagene approach, we used discriminant function analysis (DFA) to obtain a classification model predicting whether a tumor belongs to the LumE^low^ or to the non-LumE^low^ group, but based on the expression of a small set of genes (see Section 4). DFA identifies the optimal combination and number of variables that best discriminate between predefined sample populations (e.g., low and high LumE tumors). The TCGA cohort was used to derive the DFA-based classification model, which was further validated in the Setlur, Taylor, and Glinsky datasets. Samples in each dataset were labeled based on the LumE score: “LumE^low^“group (quartile q1 with lowest values) and the “non-LumE^low^” group (quartiles q2, q3, and q4). We filtered out any gene that was not present in all the profiling platforms. Then, feature (gene) selection was performed on common genes between all 4 datasets (*n* = 5812) with fold change >1.25 or <−1.25 between the 2 groups of samples (*n* = 3432). The 10 genes with highest discriminant performance were selected and the discriminant coefficients calculated (Supplementary Figure S12). Notably, the 10 genes included in the mini-classifier were not part of the initial metagenes and were overexpressed in either LumE^low^ tumors (i.e., C3, COL4A1, CYP7A1, MORF4l2, RRM1) or non-LumE^low^ tumors (i.e., AMACR, ANXA3, IDH1, KLK3, SLC13A3). Up to 93% of samples within the TCGA were correctly classified into the corresponding groups. The 10-gene classifier model was tested in the Setlur, Taylor, and Glinsky datasets, and the percentage of correctly classified samples computed (Figure 5A). To test the performance on the Ehro dataset, lacking the gene MORF4L2, a model based on the 9 remaining genes was re-trained in TCGA samples and validated in Erho dataset (Figure 5A). Overall, the classifier gave rise to high percentages of correct sample classifications in the testing datasets, between 78% and 85%. As expected, predicted LumE^low^ tumors displayed significantly higher BasE, EndoE, and FibromE scores (Supplementary Figure S13), and poor clinical outcome in recurrence-free (TCGA) and overall (Setlur) survival analyses (Figure 5B).

The mini-classifier exhibited ability similar to the luminal metagene in identifying variation in epithelial differentiation in prostate tumors. Consistent with the luminal metagene approach, the 10-gene classifier divided into unsupervised clustering analysis primary tumors and CRPCs based on the attenuation of luminal features in CRPCs (Figure 5C). Adeno-CRPCs and NE-CRPCs were also divided by unsupervised clustering into distinct subgroups based on the mini-classifier predictions (Figure 5D). Thus, the 10-gene classifier is an accurate surrogate of the luminal metagene for identifying phenotypic changes in tumor samples at different stages of the disease.

**Figure 6:**
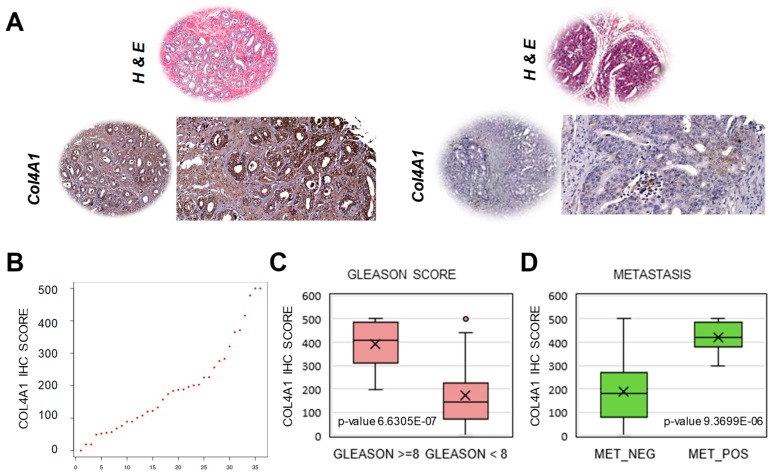
Orthogonal validation of low luminal marker COL4A1 in hormone-naïve primary prostate tumors. (**A**) COL4A1 protein was assessed by immunohistochemistry on a tissue microarray (TMA) containing primary prostate tumor samples. Representative images of high (left) and low (right) COL4A1 intensity staining. Magnification, 4× (left and upper images) and 20× (right image). (**B**) Distribution of COL4A1 immunohistochemistry (IHC) scores in primary tumors (*n* = 46). (**C**,**D**) Significant association of COL4A1 IHC staining with Gleason score (**C**) and metastasis (**D**) in primary prostate tumors.

To support our findings, we sought to provide an independent validation by assessing protein expression of classifier genes by immunohistochemistry (IHC) on primary prostate cancers. The purpose was to determine whether variations of epithelial cell differentiation with attenuated luminal phenotype could be detected by performing IHC and verify the association with clinical and pathological parameters. Among the 10 genes in the classifier, COL4A1 was significantly higher in low LumE tumors and, because of the availability of good antibodies for IHC, was selected for further orthogonal validation in an independent set of hormone-naïve primary prostate tumors (*n* = 46) with clinical and follow-up data (Figure 6A). COL4A1 immuno-staining in the tissue microarray was scored by three independent pathologists. The analysis revealed a considerable variance in the IHC score within the patient cohort, with approximately 10% of tumors with very high COL4A1 IHC score indicative of low luminal phenotype (Figure 6B). Notably, tumors with high level of COL4A1 were highly significantly associated with greater (≥8) Gleason score (Figure 6C). Moreover, a high COL4A1 IHC score in patients with primary tumors was significantly associated with metastasis within five years from diagnosis (Figure 6D). These data were consistent with the association between attenuated luminal phenotype and adverse clinical behavior. Thus, COL4A1 immunostaining provided an independent complementary validation of the classifier approach for predicting clinical behavior in patients.

### 2.5. Application of Luminal Metagene to Mouse Experimental Models

**Figure 7:**
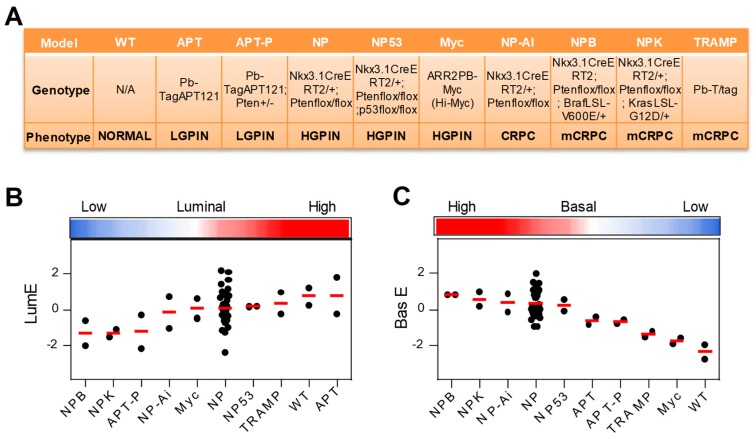
Application of metagene enrichment to genetically engineered mouse models (GEMMs) of prostate cancer. (**A**) Genotype and phenotypes of mouse models included in the study. WT, normal prostate; LGPIN and HGPIN, low-grade and high-grade prostate intraepithelial neoplasia; PC, prostate cancer; CRPC, castration-resistant prostate cancer; mCRPC, metastatic CRPC. For details see Supplementary Figure S14B,C LumE (**B**) and BasE (**C**) score distribution in mouse models.

Multiple GEMMs of prostate cancer are currently available and represent a valid resource for studying the disease [28]. To determine whether GEMMs reproduced a similar variance of the luminal phenotype as seen in human tumors, we applied our metagenes to published transcriptomic data from wild type mice and GEMMs [29]. These mouse models recapitulated different pathological stages from prostate intraepithelial neoplasia (PIN) to adenocarcinoma, CRPC, and metastatic prostate cancer (Figure 7A and Supplementary Figure S14). Interestingly, we found that more aggressive GEMMs, such as the NPB, NPK, and APT-P mice with compounded genetic alterations of oncogenes (e.g., *K-Ras, B-Raf*) and tumor suppressor genes (e.g., *Pten*, *Nkx3.1*), displayed significantly lower LumE values compared to less aggressive mouse models and normal (WT) prostate (Figure 7B). Variance also was observed in BasE scores, with NPB and NPK mice exhibiting the highest mean scores (Figure 7C). Interestingly, NPB and NPK mice with the lowest LumE score and the highest BasE score are highly metastatic, sustaining the link between low LumE and aggressive behavior. Consistently with the data in human samples, mouse tumors with lower LumE score displayed higher BasE, EndoE, and FibromE scores, resembling the human LumE^low^ tumors (Supplementary Figure S15). Thus, these data supported the notion that the luminal metagene applies also to mouse models and a low LumE score identifies invasive and metastatic models similar to human tumors.

### 2.6. Luminal Metagene and Therapeutic Response in Human and Mouse Experimental Models

**Figure 8:**
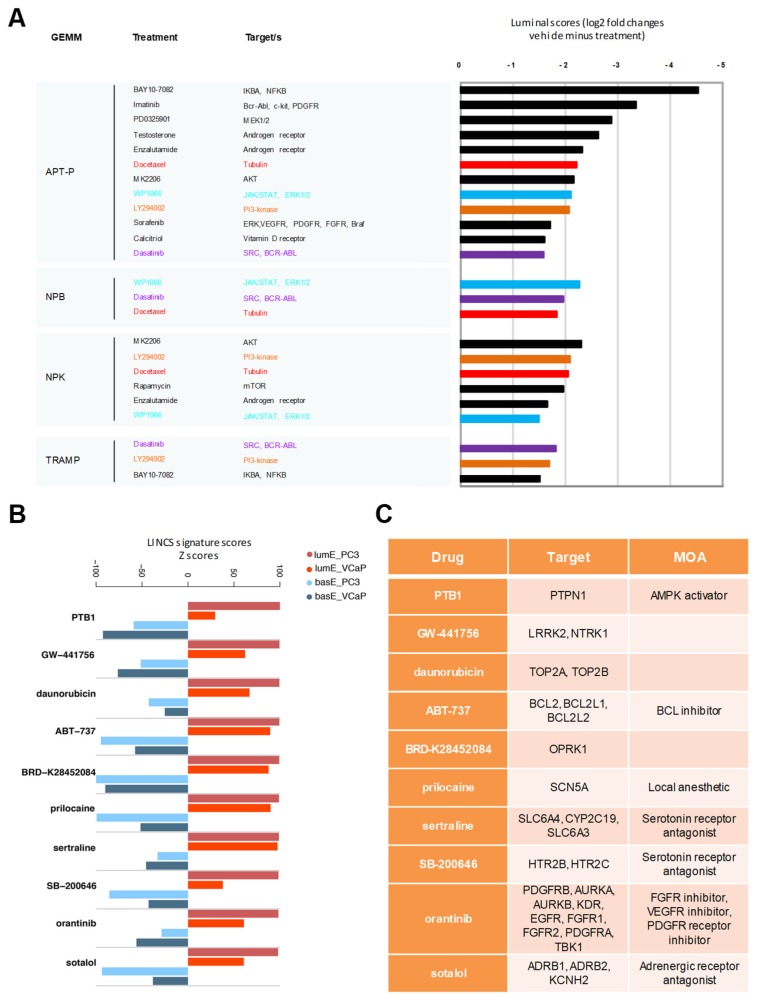
Identification of compounds enhancing luminal metagene enrichment. (**A**) Compounds increasing luminal metagene scores (≥2-fold change) in the indicated mouse models. (**B**) Top ranking compounds modifying luminal and basal enrichment scores consistently in VCaP and PC3 cells. (**C**) Targets and mechanism of action (MOA) of drugs enhancing luminal scores in both VCaP and PC3 cells. Library of Integrated Network-Based Cellular Signatures (LINCS) signature enrichment scores were defined by CLUE algorithm.

Assessing the luminal metagene score may permit monitoring the phenotypic changes induced by treatments and eventually inform on the type of biological and therapeutic response. The implemented therapy could influence the cell differentiation state and be reflected in changes in the luminal metagene enrichment. Furthermore, a shift toward higher or lower luminal enrichment might predict increased or decreased susceptibility to AR-targeted therapies. To verify this possibility, we used published transcriptomic data from GEMMs treated with anticancer drugs [29]. Our hypothesis was that treatment could influence the degree of luminal differentiation of the tumors, which might predict a therapeutically beneficial effect. Using pre- and posttreatment RNA-seq data, we computed the changes in LumE score after treatment in NPB, NPK, APT-P, and TRAMP mice. This bioinformatics analysis revealed various drugs that affected positively the luminal enrichment score shifting tumor cells to a more differentiated state (Figure 8A). We focused on compounds that gave the most frequent and consistent changes across these experimental models, aware of the bias and noise intrinsic in this type of analysis. Interestingly, in all three models (NPB, NPK, and APT-P) with the lowest starting LumE score, WP1066 (JAK/STAT inhibitor) and docetaxel (anti-tubulin drug) increased significantly the LumE score. Dasanitib (Src/Bcr-Abl inhibitor) and LY294002 (PI3 kinase inhibitor) produced a shift to higher LumE scores in three of the four models (Figure 8A).

To provide additional support to these findings, we consulted publicly available transcriptomic data for two prostate cancer cell lines (PC3 and VCaP), which had been profiled before and after treatment with many drugs/compounds in the context of the Library of Integrated Network-Based Cellular Signatures (LINCS) project (http://www.lincsproject.org/). Using the transcriptomic data for the entire library of compounds in the LINCS project, we calculated the corresponding LumE score in PC3 and VCaP cells for each compound in the database. Interestingly, compounds that scored positive in GEMMs modulated the luminal phenotype also in the cell lines (Supplementary Figure S16), although the direction of the changes was not always consistent in the two cell models (e.g., dasanitib and LY294002). This likely reflected differences in cell-specific factors, which will require further investigation. Indeed, PC3 cells, which are AR-independent and have relatively lower LumE score, correspond more to the aggressive mouse models examined here.

Interestingly, applying the metagene approach we identified additional compounds that consistently increased LumE or decreased BasE scores in PC3 and VCaP cells (Supplementary Table S6). Figure 8B shows the top 10 compounds that induced changes in LumE and BasE scores consistently in both cell lines. Notably, the list included compounds with very diverse targets, from TOP2A/B, AMPK, BCL2, FGFR-, and EGFR-associated kinases to serotonin and adrenergic receptors (Figure 8C). Although the mechanistic link between the molecular targets and the predicted effect on epithelial cell differentiation is unclear, the possibility of using these drugs to promote a more differentiated phenotype and perhaps increase the efficacy of AR-directed therapy is intriguing and certainly worth verifying experimentally.

## 3. Discussion

The genetic and molecular heterogeneity of prostate cancer is emerging as an important factor influencing clinical evolution, prognosis, and treatment efficacy. In this study, we provide novel insights into the phenotypic heterogeneity of primary and metastatic prostate tumors. We show that probing the divergence of epithelial cell features can inform on the properties of the individual tumors and reflect the clinical behavior of the disease. To achieve this objective, we constructed metagenes that dissected the bulk transcriptomic data on the basis of intrinsic prostate cell-specific features. Specifically, we found that the degree of luminal epithelial enrichment reflected the level of dedifferentiation, metastatic potential, androgen-dependency, and sensitivity to AR-directed therapies. Deviation toward an attenuated luminal phenotype depicted tumors prone to clinical adverse behavior and ineffective response to ADT.

Recent studies have shown that both luminal and basal-type progenitor/stem cells can generate prostate adenocarcinomas, which in most cases exhibit a predominant luminal phenotype with AR positivity and dependency on androgen stimulation [30]. We hypothesized that the degree of retention or divergence from the normal epithelial progenitor/stem cells may alter the context and confer distinctive properties to the individual tumors. Accordingly, our strategy was to define gene signatures or metagenes that could assess the relative representation of the diverse cell types in the prostate gland and estimate the divergence of the tumor cells from the normal state. We found that the luminal metagene was the most effective at identifying normal prostate from other normal tissues and at discriminating prostate cancers from other tumor types. Thus, the luminal metagene contained prostate cell-specific features, identified prostate tissue samples, and gave information on the state of differentiation of prostate epithelium in both the normal and pathological conditions. Despite the fact that basal cell signatures have been proposed as tumor classifiers [6], we found that the basal metagene derived from normal basal epithelial cells was less informative in this context. The basal cell signature was less able to discriminate prostate tissues from other tissues both in normal and pathological conditions. Thus, basal epithelial cells are less tissue-specific and may comprise a pool of progenitor/stem cells with shared characteristics in other epithelial tissues.

According to a precision medicine approach, specific tumor features should guide management and treatment of patients. Therefore, there is great interest in tools that might predict clinical evolution and response to treatment in individual cases. Various types of gene classifiers have been proposed to formulate predictions on clinical outcome in prostate cancer patients [6,7,31]. In a recent study, a gene classifier developed for breast cancer, PAM50, was applied to primary prostate cancers [5]. In analogy with breast cancers, primary prostate tumors were divided into three subgroups: luminal A, luminal B, and basal. Unlike the breast cancer subtypes, luminal B prostate cancers had the poorest prognosis and were concomitantly predicted to respond better to hormonal therapy, suggesting intrinsic differences between the predicted subtypes of breast and prostate cancer.

While the presence of luminal and basal features appears a unifying biological concept that could be applied to all epithelial tumors, our goal was to provide a more prostate-specific classifier that would take into account the subtle divergences between normal and pathological states. Indeed, we successfully constructed a gene signature that estimated quantitatively the degree of luminal enrichment in individual prostate tumors and resulted in homogeneous and reproducible subgrouping of prostate tumors across multiple patient cohorts. In this context, tumors with low luminal enrichment scores displayed increased recurrence rates and concomitantly attenuated AR signaling. Low luminal enrichment was associated with progression to castration-resistance. Intriguingly, however, CRPCs comprised a small fraction of tumors that retained a relatively high LumE score, suggesting residual responsiveness to AR signaling in these individual cases. Furthermore, our metagene approach detected differences in the degree of luminal enrichment in metastases at different organ sites, which might also predict loss or retention of susceptibility to AR-directed therapies. Consistently, bone metastases exhibited a higher luminal score than metastases to lymph node, lung, and liver.

An important aspect is the possibility to predict the potential benefits of therapeutic interventions in terms of biological response beyond and independently of measurable clinical responses. This would be particularly useful in borderline cases where it is important to balance the benefits and risks of treatment intensification. We hypothesized that the assessment of the degree of luminal differentiation based on the metagene score could serve such purpose by evaluating the evolutionary stage of the tumor and predicting the biological impact of a specific treatment. Concerning the response to AR-directed therapies, our data suggest that primary and metastatic prostate cancers with low LumE score might be less likely to respond to the treatment and would rather benefit from alternative strategies. Collectively, these results support the possibility of using the metagene approach to assess phenotypic changes as a biological readout of treatment efficacy in experimental models. This method can also give insights for designing new therapeutic strategies and testing new drug combinations in model systems prior to clinical implementation. Furthermore, a similar approach could be applied to characterize biologically the response to treatments in patients.

Intriguingly, application of our metagene approach to GEMMs and human cell lines provided also insights into novel strategies that could revert prostate tumors to a more luminal state and perhaps enhance or restore sensitivity to AR-based therapies. Many of the top-ranking compounds that favored a luminal phenotype in both GEMMs and cell lines are drugs already in clinical use or in clinical trials in cancer patients. Notably, a series of compounds with non-canonical mechanisms of anticancer activity and molecular targets (e.g., adrenergic receptor antagonists) but with preclinical evidence of efficacy in prostate cancer [32,33,34] emerged from our analysis. The finding of neurotransmitter receptor antagonists as potential modulators of luminal epithelial differentiation is particularly intriguing in light of the emerging role of innervation and neurotransmitter signaling in promoting tumor growth and establishing a pro-tumorigenic microenvironment [35,36,37]. Future studies will have to validate these hypotheses in preclinical models and assess the efficacy of these novel treatment modalities in clinical trials. If confirmed in preclinical models, these concepts could be readily tested in patients.

## 4. Methods

### 4.1. Metagene Discovery

Oudes datamatrix, containing high-quality hybridizations from four prostate cell types, was used to extract differentially expressed genes [10]. Gene expression profiles were generated using Human Genome U133 Plus 2.0 GeneChips (Affymetrix, Santa Clara, CA, USA). Five separate biological replicates of each sorted cell population were assayed to produce a data set of 20 chips. The GeneChips were prepared, hybridized, and scanned according to the protocols provided by Affymetrix as previously described [10]. Briefly, raw CEL files were retrieved and signal intensity obtained upon RMA processing using RMAExpress software. RLE/NUSE plots were calculated to check for quality control raw files. Differential expression analysis was performed using *t*-test to extract genes overexpressed in each cell compartment versus the others (i.e., (i) CD104/ITGB4 versus non-CD104/ITGB4; (ii) CD26/DPP4 versus non CD26/DPP4; (iii) CD49a/ITGA1 versus CD49a/ITGA1; and (iv) CD31/PECAM1 versus CD31/PECAM1). Genes in each cell-specific metagene were selected based on significance (FDR < 0.05, *p*-values based on permutations) and high T-values, giving rise to 138 genes highly representative of each cell population (Supplementary Table S1).

### 4.2. Patient Datasets

Main cohorts of prostate cancer transcriptome datasets are listed in Supplementary Table S2. Gene expression data matrices were downloaded from public repositories (Setlur, Taylor, TCGA-PRAD, Grasso, Kumar, Cai and Best), obtained from authors (Glinsky) or recalculated from raw data (Ehro). Gene expression data matrices from the individual dataset cohorts were loaded into the ssGSEA module from the GenePattern software. The output enrichment values were transformed using z-log_2_ standardization to allow direct comparison of enrichment scores from different dataset cohorts. z-log_2_ standardization transformed unlogged enrichment scores so that the mean value equals 0 and standard deviation equals 1. Scaling and centering the data with z-log_2_ normalization strategy avoids outlier issues and allows each feature to have equal relevance. In addition, ssGSEA methods do not require the datasets to have the same expression distribution, since the algorithm computes the enrichment score based on the gene rank, making the results easily comparable and similarly distributed.

### 4.3. Metagene Scores Calculation

Single-sample gene set enrichment analysis (ssGSEA) [38] was applied to calculate enrichment scores of each of the four metagenes in each sample. Each ssGSEA enrichment score represents the degree to which the genes in a particular metagene are coordinately up or downregulated within a sample. ssGSEA was computed in the prostate datasets using default settings of the module version from GenePattern software (Broad Institute). The output of each dataset consists of enrichment values per sample per metagene. Scores were further transformed into z-log_2_.

### 4.4. Patient Stratification for Kaplan-Meier Curves

Patients with coupled gene expression and clinical follow-up data were allocated in 3 groups depending on Luminal enrichment (LumE) score values: LumE^low^, quartile q1; LumE^intermed^, quartiles q2 and q3; and LumE^high^, quartile q4. Kaplan–Meier curves were obtained using Statistical Package for the Social Sciences (SPSS) 17.0 (IBM, New York, NY, USA).

### 4.5. Time Series Analysis

Massie dataset [39] was used to analyze genes regulated by androgen in prostate cancer. Briefly, LNCaP cells were grown for 72 h in Roswell Park Memorial Institute (RPMI) 1640 Medium supplemented with 10% charcoal dextran stripped fetal bovine serum (FBS). Samples harvested for transcriptome analyses comprised: (i) 3 time zero samples; (ii) 10 vehicle (ethanol) control samples taken at 2, 4, 8, 12, and 24 h in duplicate; (iii) 36 androgen (R1881) treated samples taken every 30 min for 4 h then every hour until 24 h following treatment (with replicates at 1, 2, 4, 8, 12, 16, 20, and 24 h). Expression data were downloaded from gene expression omnibus GEO (GSE18684) and metagene scores calculated using ssGSEA (see above). Significance of correlation between time and changes in metagene score values was calculated with Fisher’s Kappa statistics using the JMP software (SAS Institute, Cary, NC, USA).

### 4.6. Differential Expression between LumE^low^ and Non-LumE^low^ Human Primary Tumors

GSEA was computed between LumE^low^ (quartile q1) and non-LumE^low^ (quartile q2, q3, and q4) samples using c5.bp genesets from the Molecular Signature Database.

### 4.7. Development of LumE^low^ Gene Mini-Classifier

Discriminant function analysis (DFA) is a multivariate technique for describing a mathematical function that will distinguish among predefined groups of samples. Specifically, we used the classical discriminant analysis or linear discriminant analysis. TCGA-PRAD dataset was used as training dataset to derive the DFA-based classification model. Preliminary filtering was performed to remove genes not detectable in all platforms used in the different datasets. Validation was then performed in the Setlur, Taylor, and Glinsky datasets. Each sample in the dataset was previously labeled depending on the LumE score: samples belonging to the first quartile (q1, 25th quartile with lowest values) of the LumE scores were assigned to the “LumE^low^” group; samples with LumE scores belonging to quartiles q2, q3, and q4, were considered “non-LumE^low^” group. Feature selection was performed on common genes between all 4 datasets (*n* = 5812) with fold change >1.25 or <−1.25 between the 2 groups of samples (*n* = 3432). Forward stepwise variable selection was used, such that the 10 genes with highest discriminant performance were selected: AMACR, ANXA3, IDH1, KLK3, SLC13A3, C3, COL4A1, CYP7A1, MORF4L2, and RRM1. Discriminant coefficients were calculated for these genes, so that positive or negative coefficient values were associated with genes overexpressed or underexpressed in non-LumE^low^ samples, respectively. DFA analysis was done using the JMP software (SAS Institute).

### 4.8. Immunohistochemistry

A tissue microarray (TMA) containing 46 primary prostate tumors was constructed as previously described [40]. The Independent Ethical Committee of “Policlinico Tor Vergata” approved the study protocol (Reference number #129.18). All experimental procedures were carried out according to the Code of Ethics of the World Medical Association (Declaration of Helsinki). Informed consent was obtained from all patients prior to surgery. Specimens were handled and procedures carried out in accordance with the approved guidelines. Paraffin-embedded surgical samples were obtained to perform histological classifications and tissues microarray (TMA) construction. Serial TMA paraffin sections were used for immunohistochemistry analysis. Staining was performed using anti-Collagen IV alpha1 (COL4A1) monoclonal antibody (Sigma Aldrich, Milan, Italy SAB4200709). Antigen retrieval was performed on 4 μm thick paraffin sections using EDTA citrate pH 7.8 buffers for 30 min at 95 °C. Sections were then incubated for 1 h at room temperature with the mouse monoclonal antibody anti-COL4A1 (clone col-94; 8 µg/mL). Washings were performed with PBS/Tween20 pH 7.6. Reactions were revealed by HRP-labeled-streptavidin and a biotinylated anti-pan secondary antibody-DAB Detection Kit (UCS Diagnostic, Rome, Italy). IHC staining was scored independently by two expert pathologists to establish the degree of staining positivity in each sample.

### 4.9. Statistical Analysis

Receiver operator characteristic (ROC) curve was calculated using SPSS Statistics 17.0 and JMP software (SAS Institute, Cary, NC, USA). ROC plots graphically illustrate the performance of binary classifier system, that is, a parameter that distinguished between two groups of samples, like normal/tumor or prostate cancer/non-prostate cancer. The area under the curve (AUC) is a measure of how well the parameter distinguish between the two groups, with AUC values near 1 indicating good discriminating power of the variable and values near 0 no or limited power. *T*-test to obtain metagenes was performed with MultiExperiment Viewer (MeV). Times series analysis and DFA were done using JMP software (SAS Institute, Cary, NC, USA). GenePattern webserver was used to obtain ssGSEA scores for custom metagenes, as well as standard GSEA.

### 4.10. Impact of Drug Treatments in GEMMs and Human Cancer Cell Lines

Transcriptomic data from control and drug-treated prostate cancer GEMMs were retrieved from Aytes et al. [29] (GSE53202) (Supplementary Table S2). Metagene scores were calculated before and after treatment using ssGSEA. Drugs inducing an increase of LumE score >2 were listed. Transcriptomic data from PC3 and VCaP cells before and after drug treatment with drug compounds were retrieved from the LINCS database. Increase in LumE score was assessed using CLUE enrichment algorithm (Cluster Expander of Compound Graphs). Compounds inducing a positive change in LumE score and/or a negative change in BasE were reported.

## 5. Conclusions

This study describes an innovative approach based on prostate-specific gene signatures for discriminating high-risk prostate tumors and identifying alternative therapeutic strategies to improve treatment efficacy and clinical outcome in prostate cancer patients. Using this approach, we observed a distinct subgroup of primary tumors that have low luminal features and increased risk of recurrence under androgen deprivation therapy. We derived a 10-gene classifier that could be applied in clinical studies to identify low luminal prostate tumors and support clinical management of the patients. Compounds able to revert the low luminal status provide a novel area of investigation and may represent a new strategy against aggressive tumors.

## Supplementary Materials

The following are available online at https://www.mdpi.com/2072-6694/12/1/176/s1. Figure S1: Overall experimental plan for extraction of prostate cell-type specific gene expression signatures (metagenes) and application to multiple prostate cancer datasets, Figure S2: Prostate cell type-specific metagenes and enrichment in clinical tumors. Figure S3: Prostate cell type-specific metagenes and enrichment in clinical tumors, Figure S4: Descriptive statistics of metagenes enrichment scores are similar between primary prostate cancer datasets, Figure S5: Prostate-specific metagene enrichment in normal and cancer samples, Figure S6: Enrichment of BasE, EndoE and FibromE scores in low LumE tumors in the TCGA dataset, Figure S7: GSEA enrichment plots with prostate specific genesets, Figure S8: Relationship between AR signaling and luminal metagene, Figure S9: Basal, endothelial and fibromuscular enrichment scores in primary tumors and CRPCs, Figure S10: Distribution of basal, endothelial and fibromuscular metagene scores in primary tumors and CRPCs from the Grasso dataset, Figure S11: Basal, endothelial and fibromuscular enrichment scores in metastatic prostate tumors at distinct organ sites, Figure S12: Genes included in the 10-gene classifier and their relative coefficients, Figure S13: The 10-gene classifier discriminates in the TCGA dataset LumE low and non-LumE low primary tumors with differential enrichment of prostate-specific metagenes, Figure S14: Genetically modified mouse models (GEMMs) of prostate cancer with published transcriptomic data used in this study, Figure S15: Differential enrichment of basal (BasE), endothelial (EndoE) and fibromuscular (FibroE) metagenes in genetically engineered mouse models (GEMMs) of prostate cancer in relation to their LumE low or non-LumE low status, Figure S16: Top ranking drugs modulating luminal metagene scores in both mouse models and cell lines, Table S1A: Luminal metagene, Table S1B: Basal metagene, Table S1C: Fibromuscular stroma metagene, Table S1D: Endothelial stroma metagene, Table S2: Gene expression datasets included in the study, Table S3: Metagene scores in Taylor dataset, Table S4: Normalized enrichment scores (NES) in Taylor and TCGA datasets, Table S5: GSEA pathways enriched in low LumE tumors, Table S6A: Target and MOA of top 30 compounds in VCaP, Table S6B: Target and MOA of top 30 compounds in PC3, Table S6C: Top 10 compounds common in PC3 and VCaP.
