# Erratum: Mapelli, S.N., et al. A Novel Prostate Cell Type-Specific Gene Signature to Interrogate Prostate Tumor Differentiation Status and Monitor Therapeutic Response (Running Title: Phenotypic Classification of Prostate Tumors). *Cancers* 2020, *12*, 176

**DOI:** 10.3390/cancers12102971

**Published:** 2020-10-14

**Authors:** Sarah N. Mapelli, Domenico Albino, Maurizia Mello-Grand, Dheeraj Shinde, Manuel Scimeca, Rita Bonfiglio, Elena Bonanno, Giovanna Chiorino, Ramon Garcia-Escudero, Carlo V. Catapano, Giuseppina M. Carbone

**Affiliations:** 1 Institute of Oncology Research (IOR), Università della Svizzera italiana (USI), 6500 Bellinzona, Switzerland; Sarah.mapelli@ior.usi.ch (S.N.M.); Domenico.albino@ior.usi.ch (D.A.); Dheeraj.shinde@ior.usi.ch (D.S.); 2 Swiss Institute of Bioinformatics (SIB), 1015 Lausanne, Switzerland; 3 Laboratory of Cancer Genomics, Fondazione Edo ed Elvo Tempia Valenta, 13900 Biella, Italy; maurizia.mellogrand@fondazionetempia.org (M.M.-G.); giovanna.chiorino@fondazionetempia.org (G.C.); 4 Department of Biomedicine and Prevention, University of Rome “Tor Vergata”, 00133 Rome, Italy; manuel.scimeca@uniroma2.it (M.S.); Rita.bonfiglio@gmail.com (R.B.); elena.bonanno@uniroma2.it (E.B.); 5 Molecular Oncology Unit, CIEMAT, 28040 Madrid, Spain; 6 Biomedicine Research Institute, Hospital 12 octubre, 28040 Madrid, Spain; 7 Centro de Investigación Biomédica en Red de Cáncer (CIBERONC), 28040 Madrid, Spain; 8 Department of Oncology, Faculty of Biology and Medicine, University of Lausanne, 1011 Lausanne, Switzerland

There is an error in the title of the paper [1]. The authors thus wish to make the following correction to this paper [1]:

Change the title from “A Novel Prostate Cell Type-Specific Gene Signature to Interrogate Prostate Tumor Differentiation Status and Monitor Therapeutic Response (Running Title: Phenotypic Classification of Prostate Tumors)” to “A Novel Prostate Cell Type-Specific Gene Signature to Interrogate Prostate Tumor Differentiation Status and Monitor Therapeutic Response”. 

We apologize for this error and state that the scientific conclusions are unaffected. The original article has been updated.
