# Supplementary material for: A Novel Prostate Cell Type-Specific Gene Signature to Interrogate Prostate Tumor Differentiation Status and Monitor Therapeutic Response (Running Title: Phenotypic Classification of Prostate Tumors)
Source: Cancers (Basel). 2020 Jan 10;12(1):176. doi: 10.3390/cancers12010176 (PMC7016595; doi:10.3390/cancers12010176)
Supplement: Supplementary file 1 [file cancers-12-00176-s001.zip › SUPPLEMENTARY/cancers-678534-SUPPLEMENTARY FINAL.docx]

**Supplementary Materilas**


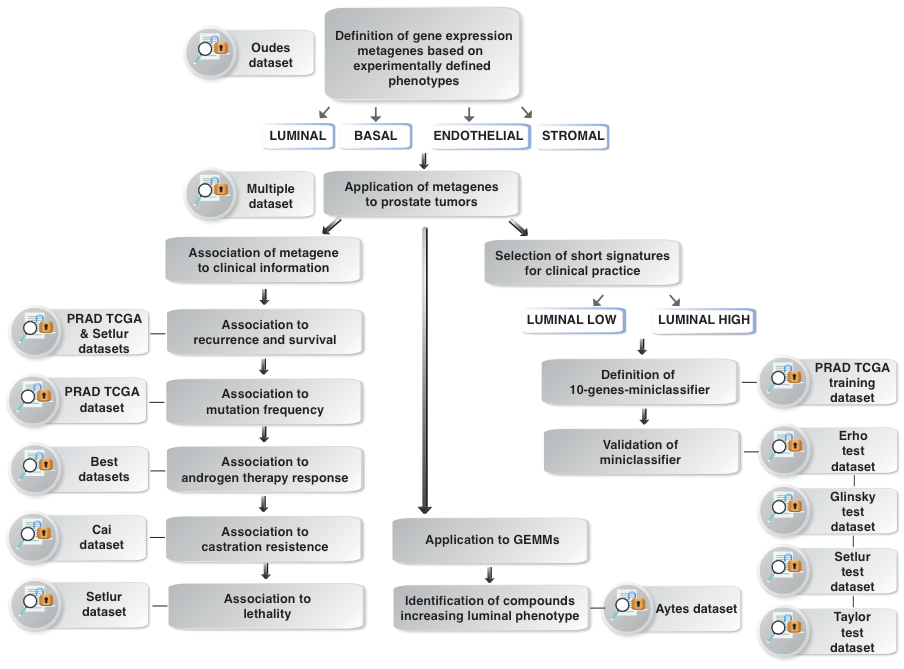


**Figure S1.** Overall experimental plan for extraction of prostate cell-type specific gene expression signatures (metagenes) and application to multiple prostate cancer datasets. Metagenes for luminal, basal, fibromuscular and endothelial prostatic cells were obtained. Single-sample enrichment of each metagene was calculated in different prostate cancer studies to determine possible association with clinical outcome, genetic alterations, response to androgen therapy, resitance to castration, and lethal disease. Metagenes enrichment was also calculated in genetically modified mouse models (GEMMs) of prostate cancer in order to find associations with tumor phenotype and response to treatment. A 10-gene mini-classifier was also obtained and validated to predict luminal metagene enrichment in clinical samples.

**Taylor**

**Setlur**

**TCGA**


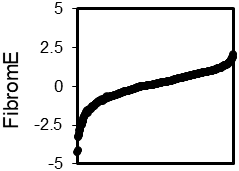

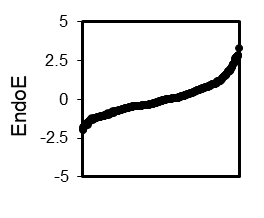

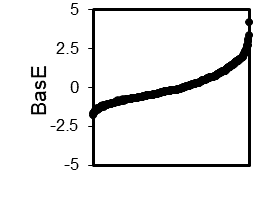

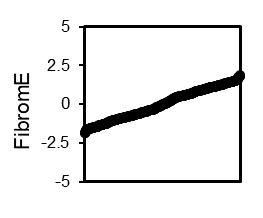

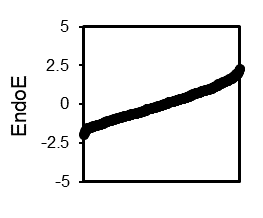

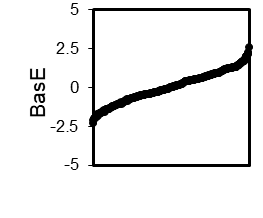

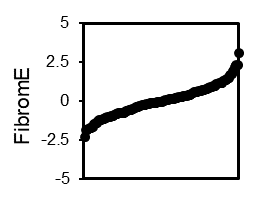

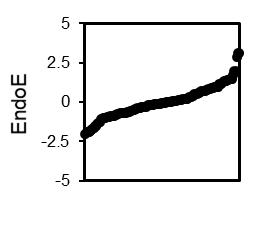

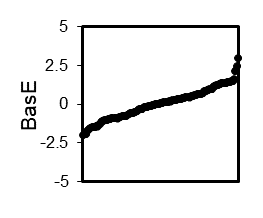


**A**

**B**

**C**

**Basal**

**Fibromuscular**

**Endothelial**

**Figure S2.** Prostate cell type-specific metagenes and enrichment in clinical tumors. Basal (**A**), fibromuscular (**B**) and endothelial (**C**) metagene enrichment scores (BasE, FibromE and EndoE, respectively) using single sample GSEA (ssGSEA) approach in primary prostate cancer from the Taylor, Setlur and TCGA datasets (see Supplementary Table 2). Samples were ordered with increasing score values. Metagene enrichment scores are shown at the z-log_2_ scale.

**A**

**B**

Taylor

Taylor

Setlur

Setlur

TCGA

TCGA

Enrichment score

Taylor

Taylor

Setlur

Setlur

TCGA

TCGA

Enrichment score

**Figure S3.** Prostate cell type-specific metagenes and enrichment in clinical tumors. Enrichment scores for luminal and basal (**A**), and fibromuscular and endothelial (**B**) metagenes (LumE, BasE, FibromE and EndoE, respectively) determined by single sample GSEA (ssGSEA) in primary tumors from distinct datasets (Taylor, Setlur and TCGA). Metagene enrichment scores are shown at the z-log_2_ scale. Median values and the +2 and -2 score range are indicated.

**Figure S4.** Descriptive statistics of metagenes enrichment scores are similar between primary prostate cancer datasets. Various comparative parameters (minimum, 25% percentile, median, 75% percentile and maximum NES) are plotted for basal (**A**), fibromuscular (**B**) and endothelial (**C**) metagenes. Metagene enrichment scores are shown at the z-log_2_ scale.

**Figure S5.** Prostate-specific metagene enrichment in normal and cancer samples. BasE (**A**), FibromE (**B**), and EndoE (**C**) scores in normal and cancer tissue samples from TCGA-pancancer dataset. Samples are grouped by organ site and ordered from highest to lowest median value. Area under the ROC Curve (AUC) values near 1.0 (with asterisk) indicate significant discrimination between prostatic and non-prostatic tissues. Metagene enrichment scores in are shown at the z-log_2_ scale.

**Figure S6.** (**A**) Enrichment of BasE, EndoE and FibromE scores in low LumE tumors in the TCGA dataset. Patients were stratified depending on LumE score into LumE^low^ (quartile q1) and non-LumE^low^ (quartiles q2, q3 and q4). Significant differences in BasE, EndoE and FibromE scores were calculated by t-test and p-values are reported. Metagene enrichment scores are shown at the z-log_2_ scale. (**B**) Univariate Cox regression analysis in the TCGA and Setlur datasets (recurrence-free survival and overall survival, respectively) using an immune metagene score (see Methods). Immune scores were not significantly associated with prognosis in both cohorts. (**C**) Immune scores in LumE low and non-LumE low tumors in the TCGA and Setlur cohorts. Samples were grouped and analyzed as described above and relative p-values are reported.


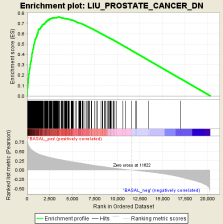

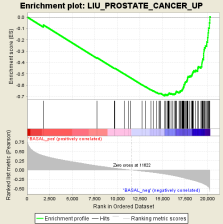

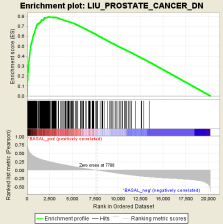

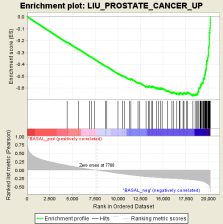


Taylor

TCGA

LIU_PROSTATE_CANCER_UP

LIU_PROSTATE_CANCER_DOWN

Higher BasE versus lower BasE

FDR<0.01

FDR<0.01

FDR<0.01

FDR<0.01

**Figure S7.** GSEA enrichment plots with prostate specific genesets. Significant differences in enrichment were observed in tumors according to their BasE scores in the two datasets examined.

**B**

Supplementary Figure 8. Relationship between AR signaling and luminal metagene. A) Behavior of LumE, BasE, FibromE and EndoE metagenes in a time series analysis in LNCaP cells treated with R1881. Transcriptomic data were retrieved from the Massie dataset (see Methods) that examined genes regulated by androgen in prostate cancer cells. LNCaP cells were treated and collected at different times to perform RNA extraction and transcriptome analysis. Significance of time-dependent changes of metagene scores was calculated using Fisher's Kappa statistic and p-values are reported. Significant changes were observed for the LumE and BasE scores. B) GSEA enrichment plots using a geneset of androgen downregulated genes (DOANE_ANDROGEN_RESPONSE_DOWN) in TCGA and Taylor datasets. Significant enrichment of the genesets is observed in tumors with higher BasE scores. C) LumE scores in primary hormone naïve (primary^HN^) versus primary castration resistant (primary^CR^) prostate tumors in Best dataset. (D) LumE scores in primary hormone-naïve (primary^HN^) versus metastatic castration resistant prostate tumors (mCRPC) in the Cai dataset. Metagene score values are shown as z-log_2_.

**A**

FibromE

-2

0

2

0

5

10

15

20

25

time (h)

p-val=0.64

EndoE

-2

0

2

0

5

10

15

20

25

time (h)

p-val=0.57

LNCaP + R1881

BasE

-2

0

2

0

5

10

15

20

25

p-val=0.028

LumE

-2

0

2

0

5

10

15

20

25

p-val=6.1x10^-5^

time (h)

time (h)


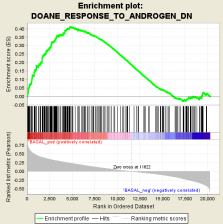

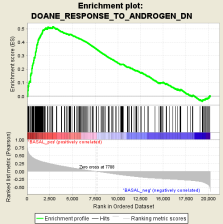


TCGA

Taylor

p-val = 0.0004

**Best**

**C**

p-val < 0.0001

**Cai**

**D**

DOANE_ANDROGEN_RESPONSE_DOWN

Higher BasE versus lower BasE

FDR<0.01

FDR<0.01

**Figure S8.** Relationship between AR signaling and lumina lmetagene. (**A**)Behavior of LumE, BasE, FibromE and EndoE metagenes in a time series analysis in LNCaP cells treated with R1881.Transcriptomic data were retrieved from the Massie data set (seeMethods) that examined genes regulated by androgen in prostate cancer cells. LNCaP cells were treated and collected at different times to perform RNA extraction and transcriptome analysis. Significance of time-dependent changes of metagene scores was calculated using Fisher's Kappa statistic and p-values are reported. Significant changes were observed for the LumE and BasEscores. (**B**) GSEA enrichment plots using a gene set of androgen downregulated genes (DOANE_ANDROGEN_RESPONSE_DOWN) in TCGA and Taylor data sets. Significant enrichment of the gene sets is observed in tumors with higher BasEscores. (**C**) LumE scores in primary hormone naïve (primaryHN) versus primary castration resistant (primaryCR) prostate tumors in Best dataset. (**D**) LumE scores in primary hormone-naïve (primaryHN) versus metastatic castration resistant prostate tumors (mCRPC) in the Cai dataset. Metagene score values are shown as z-log2.

Fibromuscular


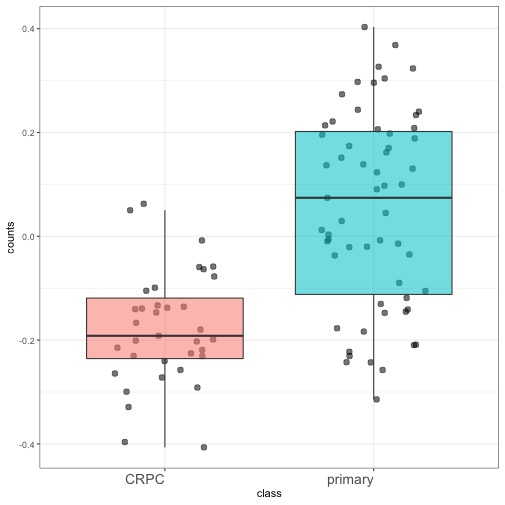


p-value = 3.575e-11

Endothelial


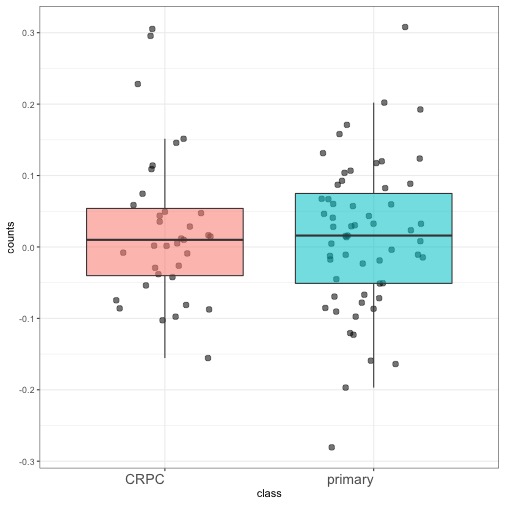


p-value = 0.6335

Basal


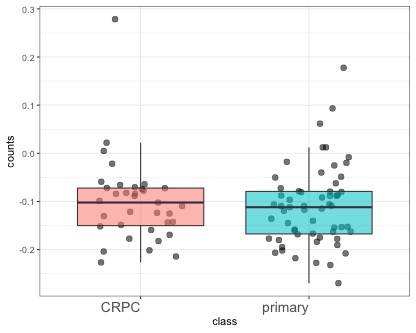


p-value = 0.5974

**Figure S9.** Basal, endothelial and fibro muscular enrichment scores in primary tumors and CRPCs. Transcriptomi cdata were retrieved from the Grasso dataset (see Supplementary Table2). Significance is indicated by p-values computed with Welch Two sample t-test.

**B**

**A**

**C**

**Figure S10.** Distribution of basal (**A**), endothelial (**B**) and fibromuscular (**C**) metagene scores in primary tumors and CRPCs from the Grasso dataset. Samples are ordered based on increasing score values for the indicated metagenes. Red, CRPCs; Black, primary prostate tumors. Note that primary and CRPC samples are randomly distributed with basal and endothelial scores. Weak clustering is observed with the fibromuscular score.

**
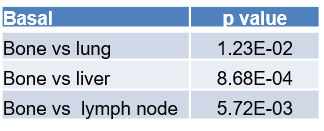

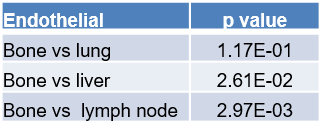

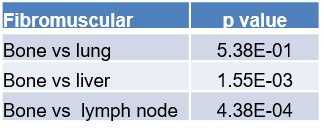
Figur**e S**11**. Basal, endothelial and fibromuscular enrichment scores in metastatic prostate tumors at distinct organ sites. Tables show p-values relative to the comparisons of each metagene scores between bone and lung, liver or lymph node metastases. *, significant differences.

Basal


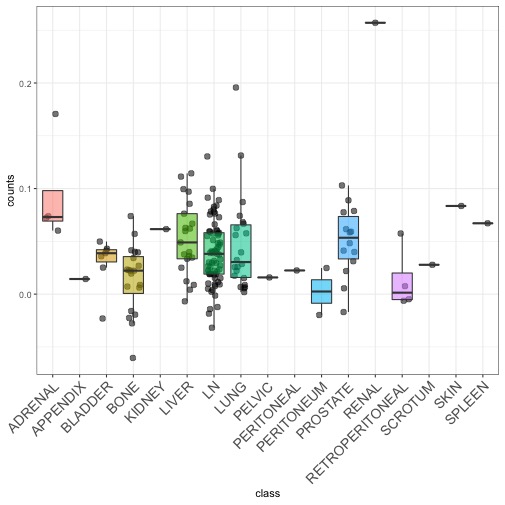


ssGSEA score

Endothelial


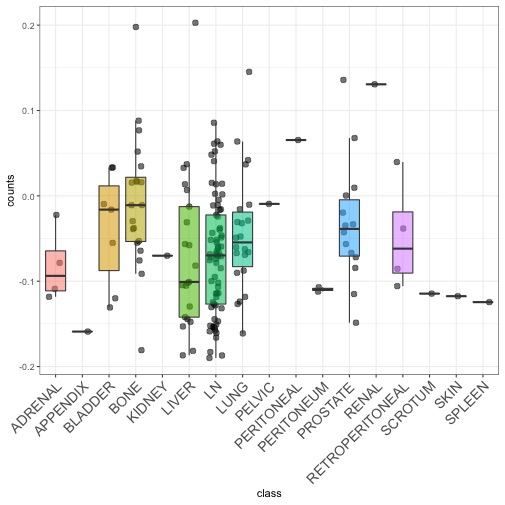


ssGSEA score

Fibromuscular


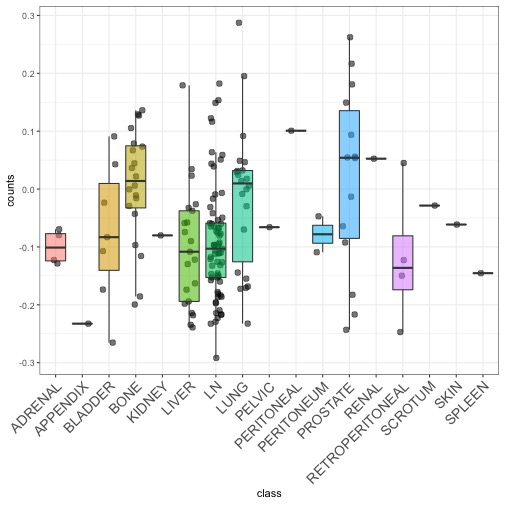


ssGSEA score


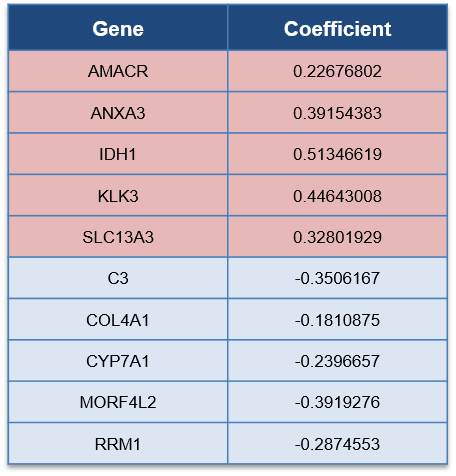


**Figure S12.** Genes included in the 10-gene classifier and their relative coefficients derived by Discriminant Function Analysis (DFA) applied to the TCGA datasets.

**Figure S13.** The 10-gene classifier discriminates in the TCGA dataset LumE low and non-LumE low primary tumors with differential enrichment of prostate-specific metagenes. Enrichemnt scores for LumE, BasE, EndoE and FibromE in the predicted groups are shown with the relative p-values calculated by t-test.


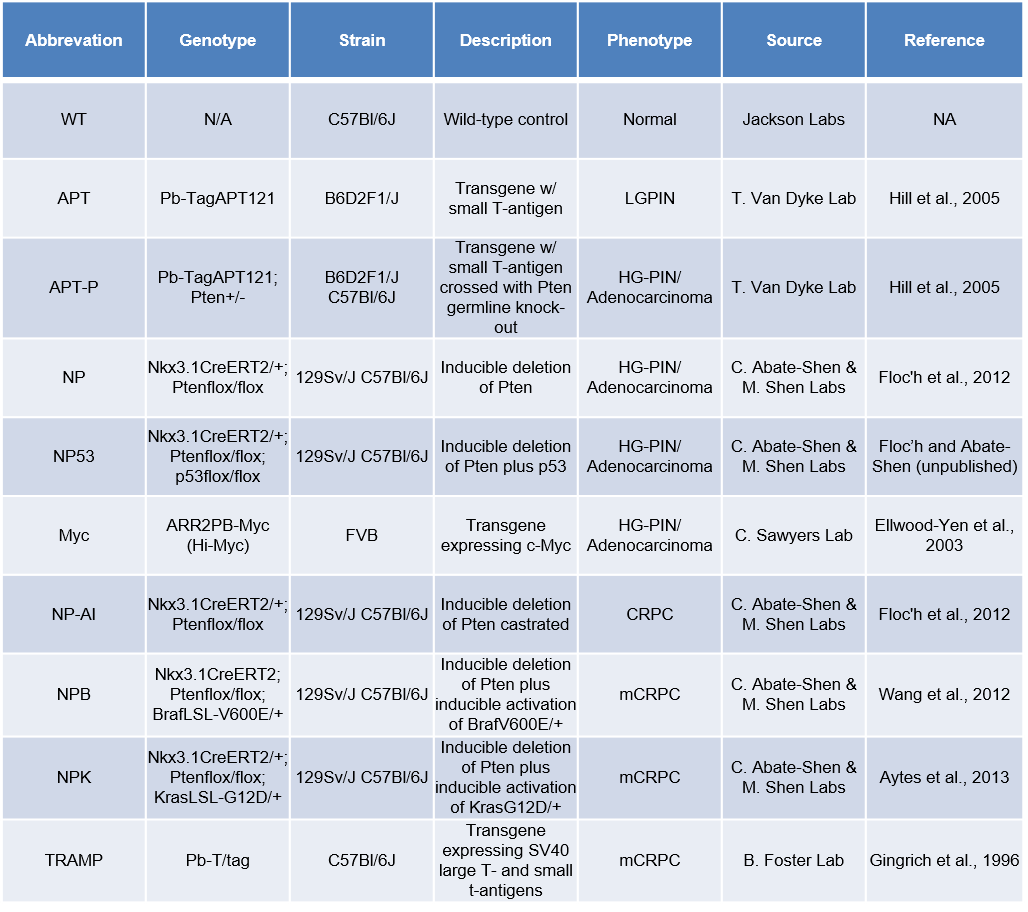


**Figure S14.** Genetically modified mouse models (GEMMs) of prostate cancer with published transcriptomic data used in this study. Genotype, phenotype, source and original references are indicated for each model.

Supplementary Figure 14. Genetically modified mouse models (GEMMs) of prostate cancer with published transcriptomic data used in this study. Genotype, phenotype, source and original references are indicated for each model.

Supplementary Figure 15. Differential enrichment of basal (BasE), endothelial (EndoE) and fibromuscular (FibroE) metagenes in genetically engineered mouse models (GEMMs) of prostate cancer in relation to their LumE low or non-LumE low status. P-values were calculated using t-test.

p-val=0.005

p-val=0.05

p-val=0.2

**Figure S15.** Differential enrichment of basal (BasE), endothelial (EndoE) and fibromuscular (FibroE) metagenes in genetically engineered mouse models (GEMMs) of prostate cancer in relation to their LumE low or non-LumE low status. P-values were calculated using t-test.

LINCS enrichment scores

Z scores

LINCS signature scores

Z scores

**Figure 16.** Top ranking drugs modulating luminal metagene scores in both mouse models and cell lines.
