# Supplementary material for: A Novel Prostate Cell Type-Specific Gene Signature to Interrogate Prostate Tumor Differentiation Status and Monitor Therapeutic Response (Running Title: Phenotypic Classification of Prostate Tumors)
Source: Cancers (Basel). 2020 Jan 10;12(1):176. doi: 10.3390/cancers12010176 (PMC7016595; doi:10.3390/cancers12010176)
Supplement: Supplementary file 1 [file cancers-12-00176-s001.zip › SUPPLEMENTARY/Supp tables_R1.pdf]

Supplementary Table 1A. Luminal metagene

Symbol	Entrez Gene Name	Location	Type(s)
ABAT	4-aminobutyrate aminotransferase	Cytoplasm	enzyme
ACSS1	acyl-CoA synthetase short-chain family member 1	Cytoplasm	enzyme
AP1M2	adaptor-related protein complex 1, mu 2 subunit	Cytoplasm	transporter
ARRDC1	arrestin domain containing 1	Cytoplasm	other
ATP2C2	ATPase, Ca++ transporting, type 2C, member 2	Cytoplasm	enzyme
ATP6V0E2	ATPase, H+ transporting V0 subunit e2	Cytoplasm	enzyme
AUH	AU RNA binding protein/enoyl-CoA hydratase	Cytoplasm	enzyme
BCAS1	breast carcinoma amplified sequence 1	Plasma Membrane	other
BDH1	3-hydroxybutyrate dehydrogenase, type 1	Cytoplasm	enzyme
BSPRY	B-box and SPRY domain containing	Cytoplasm	other
C11orf80	chromosome 11 open reading frame 80	Other	other
C19orf48	chromosome 19 open reading frame 48	Other	other
C1QTNF9B-AS1	C1QTNF9B antisense RNA 1	Cytoplasm	other
C8orf82	chromosome 8 open reading frame 82	Cytoplasm	other
CAB39L	calcium binding protein 39-like	Cytoplasm	kinase
CCDC64B	coiled-coil domain containing 64B	Other	other
CD320	CD320 molecule	Plasma Membrane	other
CD38	CD38 molecule	Plasma Membrane	enzyme
CGN	cingulin	Plasma Membrane	other
CNDP2	CNDP dipeptidase 2 (metallopeptidase M20 family)	Cytoplasm	peptidase
COBL	cordon-bleu WH2 repeat protein	Plasma Membrane	other
COL9A1	collagen, type IX, alpha 1	Extracellular Space	other
COLEC12	collectin sub-family member 12	Plasma Membrane	receptor
CPLX3	complexin 3	Nucleus	transporter
CPNE4	copine IV	Cytoplasm	other
CREB3L4	cAMP responsive element binding protein 3-like 4	Nucleus	transcription regulator
CRLS1	cardiolipin synthase 1	Cytoplasm	enzyme
CWH43	cell wall biogenesis 43 C-terminal homolog (S. cerevisiae)	Other	other
DHRS7B	dehydrogenase/reductase (SDR family) member 7B	Other	other
DMXL1	Dmx-like 1	Extracellular Space	other
DNAH5	dynein, axonemal, heavy chain 5	Cytoplasm	enzyme
DPP4	dipeptidyl-peptidase 4	Plasma Membrane	peptidase
DPYS	dihydropyrimidinase	Cytoplasm	enzyme
EMB	embigin	Plasma Membrane	other

ENPP5	ectonucleotide pyrophosphatase/phosphodiesterase 5 (putative)	Extracellular Space	enzyme
ENTPD6	ectonucleoside triphosphate diphosphohydrolase 6 (putative)	Cytoplasm	enzyme
ERBB3	erb-b2 receptor tyrosine kinase 3	Plasma Membrane	kinase
ESRP2	epithelial splicing regulatory protein 2	Nucleus	other
FAAH	fatty acid amide hydrolase	Plasma Membrane	enzyme
FAAH2	fatty acid amide hydrolase 2	Other	enzyme
FAM135A	family with sequence similarity 135, member A	Other	enzyme
FAM136A	family with sequence similarity 136, member A	Cytoplasm	other
FAM174B	family with sequence similarity 174, member B	Other	other
FAM3B	family with sequence similarity 3, member B	Extracellular Space	cytokine
FAM96A	family with sequence similarity 96, member A	Extracellular Space	other
FBP1	fructose-1,6-bisphosphatase 1	Cytoplasm	phosphatase
FOLH1B	folate hydrolase 1B	Cytoplasm	other
GGTLC1	gamma-glutamyltransferase light chain 1	Plasma Membrane	enzyme
GLB1L2	galactosidase, beta 1-like 2	Other	other
GNG4	guanine nucleotide binding protein (G protein), gamma 4	Plasma Membrane	enzyme
GPR158	G protein-coupled receptor 158	Plasma Membrane	G-protein coupled receptor
GPRC5C	G protein-coupled receptor, class C, group 5, member C	Plasma Membrane	G-protein coupled receptor
GPT2	glutamic pyruvate transaminase (alanine aminotransferase) 2	Cytoplasm	enzyme
GREB1	growth regulation by estrogen in breast cancer 1	Cytoplasm	other
HGD	homogentisate 1,2-dioxygenase	Cytoplasm	enzyme
IDH2	isocitrate dehydrogenase 2 (NADP+), mitochondrial	Cytoplasm	enzyme
INTS12	integrator complex subunit 12	Nucleus	other
KIAA1244	ARFGEF family member 3	Other	other
LAMP2	lysosomal-associated membrane protein 2	Plasma Membrane	enzyme
LCP1	lymphocyte cytosolic protein 1 (L-plastin)	Cytoplasm	other
LMAN1L	lectin, mannose-binding, 1 like	Plasma Membrane	other
LOC100129034	uncharacterized LOC100129034	Other	other
LOC100505938	uncharacterized LOC100505938	Other	other
LOC100507039			
LOC81691	exonuclease NEF-sp	Nucleus	enzyme
MARVELD2	MARVEL domain containing 2	Plasma Membrane	other
MCCC2	methylcrotonoyl-CoA carboxylase 2 (beta)	Cytoplasm	enzyme

MESP1	mesoderm posterior basic helix-loop-helix transcription factor 1	Nucleus	transcription regulator
MIEN1	migration and invasion enhancer 1	Cytoplasm	other
MRPL41	mitochondrial ribosomal protein L41	Cytoplasm	other
MRPS23	mitochondrial ribosomal protein S23	Cytoplasm	other
MUC3B	mucin 3B, cell surface associated	Cytoplasm	other
MYBPC1	myosin binding protein C, slow type	Cytoplasm	other
NCAPD3	non-SMC condensin II complex, subunit D3	Nucleus	other
NIPAL3	NIPA-like domain containing 3	Other	other
NUDT16L1	nudix (nucleoside diphosphate linked moiety X)-type motif 16-like 1	Cytoplasm	other
OAZ3	ornithine decarboxylase antizyme 3	Cytoplasm	transporter
PAOX	polyamine oxidase (exo-N4-amino)	Cytoplasm	enzyme
PCCA	propionyl CoA carboxylase, alpha polypeptide	Cytoplasm	enzyme
PDE9A	phosphodiesterase 9A	Cytoplasm	enzyme
PEBP4	phosphatidylethanolamine-binding protein 4	Cytoplasm	other
PGM3	phosphoglucomutase 3	Cytoplasm	enzyme
PLEKHH1	pleckstrin homology domain containing, family H (with MyTH4 domain) member 1	Cytoplasm	other
PPAPDC1B	phosphatidic acid phosphatase type 2 domain containing 1B	Other	phosphatase
PPAPDC2	phosphatidic acid phosphatase type 2 domain containing 2	Other	phosphatase
PPM1E	protein phosphatase, Mg2+/Mn2+ dependent, 1E	Nucleus	phosphatase
PPP2R2C	protein phosphatase 2, regulatory subunit B, gamma	Other	phosphatase
PRIM2	primase, DNA, polypeptide 2 (58kDa)	Nucleus	enzyme
PRR15L	proline rich 15-like	Other	other
RAB17	RAB17, member RAS oncogene family	Cytoplasm	enzyme
RAB3B	RAB3B, member RAS oncogene family	Cytoplasm	enzyme
RAMP1	receptor (G protein-coupled) activity modifying protein 1	Plasma Membrane	transporter
RAP1GAP	RAP1 GTPase activating protein	Cytoplasm	other
REEP6	receptor accessory protein 6	Plasma Membrane	other
RFPL2	ret finger protein-like 2	Other	other
RGS17	regulator of G-protein signaling 17	Cytoplasm	other
RLN2	relaxin 2	Extracellular Space	other
RSPH1	radial spoke head 1 homolog (Chlamydomonas)	Nucleus	other
RWDD2A	RWD domain containing 2A	Other	other
SCGB1D2	secretoglobin, family 1D, member 2	Extracellular Space	other
SEC14L2	SEC14-like 2 (S. cerevisiae)	Cytoplasm	transporter
SERPINB6	serpin peptidase inhibitor, clade B (ovalbumin), member 6	Cytoplasm	other
SLC12A8	solute carrier family 12, member 8	Other	transporter

SLC15A2	solute carrier family 15 (oligopeptide transporter), member 2	Plasma Membrane	transporter
SLC16A5	solute carrier family 16 (monocarboxylate transporter), member 5	Plasma Membrane	transporter
SLC26A3	solute carrier family 26 (anion exchanger), member 3	Plasma Membrane	transporter
SLC31A1	solute carrier family 31 (copper transporter), member 1	Plasma Membrane	transporter
SLC36A1	solute carrier family 36 (proton/amino acid symporter), member 1	Plasma Membrane	transporter
SLC38A11	solute carrier family 38, member 11	Other	transporter
SLC44A4	solute carrier family 44, member 4	Plasma Membrane	transporter
SLC52A3	solute carrier family 52 (riboflavin transporter), member 3	Plasma Membrane	other
SLC9A2	solute carrier family 9, subfamily A (NHE2, cation proton antiporter 2), member 2	Plasma Membrane	transporter
SOCS2-AS1	SOCS2 antisense RNA 1	Other	other
STAP2	signal transducing adaptor family member 2	Cytoplasm	other
STYK1	serine/threonine/tyrosine kinase 1	Cytoplasm	kinase
SULT2B1	sulfotransferase family, cytosolic, 2B, member 1	Cytoplasm	enzyme
SYNE4	spectrin repeat containing, nuclear envelope family member 4	Nucleus	other
SYT7	synaptotagmin VII	Cytoplasm	transporter
TARP	TCR gamma alternate reading frame protein	Cytoplasm	other
TM7SF2	transmembrane 7 superfamily member 2	Cytoplasm	enzyme
TMC4	transmembrane channel-like 4	Cytoplasm	other
TMC5	transmembrane channel-like 5	Cytoplasm	other
TMEM125	transmembrane protein 125	Other	other
TMEM79	transmembrane protein 79	Cytoplasm	other
TMEM87B	transmembrane protein 87B	Other	other
TPMT	thiopurine S-methyltransferase	Cytoplasm	enzyme
TRIM36	tripartite motif containing 36	Cytoplasm	other
TRPM8	transient receptor potential cation channel, subfamily M, member 8	Plasma Membrane	ion channel
TRPV6	transient receptor potential cation channel, subfamily V, member 6	Plasma Membrane	ion channel
TST	thiosulfate sulfurtransferase (rhodanese)	Cytoplasm	enzyme
TTC39A	tetratricopeptide repeat domain 39A	Other	other
TTLL7	tubulin tyrosine ligase-like family member 7	Plasma Membrane	other
UNC13B	unc-13 homolog B (C. elegans)	Cytoplasm	other
VPS25	vacuolar protein sorting 25 homolog (S. cerevisiae)	Cytoplasm	other
YIPF1	Yip1 domain family, member 1	Cytoplasm	other
ZG16B	zymogen granule protein 16B	Other	other

ZNF350	zinc finger protein 350	Nucleus	transcription regulator
ZNF613	zinc finger protein 613	Nucleus	other

Supplementary Table 1B. Basal metagene

Symbol	Entrez Gene Name	Location	Type(s)
ABI1	abl-interactor 1	Cytoplasm	other
ABLIM1	actin binding LIM protein 1	Cytoplasm	other
ATXN1	ataxin 1	Nucleus	transcription regulator
AVPI1	arginine vasopressin-induced 1	Other	other
C1orf106	chromosome 1 open reading frame 106	Other	other
CA12	carbonic anhydrase XII	Plasma Membrane	enzyme
CDCA7L	cell division cycle associated 7-like	Nucleus	other
CENPL	centromere protein L	Cytoplasm	other
CHST9	carbohydrate (N-acetylgalactosamine 4-0) sulfotransferase 9	Cytoplasm	enzyme
CLCA2	chloride channel accessory 2	Plasma Membrane	ion channel
CLCA4	chloride channel accessory 4	Plasma Membrane	ion channel
CPA6	carboxypeptidase A6	Extracellular Space	peptidase
CRNDE	colorectal neoplasia differentially expressed (non-protein coding)	Other	other
CSNK1E	casein kinase 1, epsilon	Cytoplasm	kinase
CSTA	cystatin A (stefin A)	Cytoplasm	other
CYP4B1	cytochrome P450, family 4, subfamily B, polypeptide 1	Cytoplasm	enzyme
D4S234E			
DEFB1	defensin, beta 1	Extracellular Space	other
DENND2C	DENN/MADD domain containing 2C	Other	other
DEPDC7	DEP domain containing 7	Cytoplasm	other
DSC3	desmocollin 3	Plasma Membrane	other
DUOX1	dual oxidase 1	Plasma Membrane	enzyme
EDN1	endothelin 1	Extracellular Space	cytokine
ENAH	enabled homolog (Drosophila)	Plasma Membrane	other
ENTPD7	ectonucleoside triphosphate diphosphohydrolase 7	Cytoplasm	enzyme
EPHA1-AS1	EPHA1 antisense RNA 1	Other	other
EPHA2	EPH receptor A2	Plasma Membrane	kinase
ERRFI1	ERBB receptor feedback inhibitor 1	Cytoplasm	other
F3	coagulation factor III (thromboplastin, tissue factor)	Plasma Membrane	transmembrane receptor
F5	coagulation factor V (proaccelerin, labile factor)	Plasma Membrane	enzyme
FAM110C	family with sequence similarity 110, member C	Cytoplasm	other
FAM46B	family with sequence similarity 46, member B	Other	other
FEM1B	fem-1 homolog b (C. elegans)	Nucleus	transcription regulator
FGD6	FYVE, RhoGEF and PH domain containing 6	Cytoplasm	other
FGFR2	fibroblast growth factor receptor 2	Plasma Membrane	kinase
FGFR3	fibroblast growth factor receptor 3	Plasma Membrane	kinase
FHL2	four and a half LIM domains 2	Nucleus	transcription regulator
FLJ13197			

FLRT3	fibronectin leucine rich transmembrane protein 3	Plasma Membrane	other
FOXE1	forkhead box E1	Nucleus	transcription regulator
FOXQ1	forkhead box Q1	Nucleus	transcription regulator
FRMD6	FERM domain containing 6	Cytoplasm	other
FRMD6-AS1	FRMD6 antisense RNA 1	Other	other
GABRP	gamma-aminobutyric acid (GABA) A receptor, pi	Plasma Membrane	ion channel
GAD1	glutamate decarboxylase 1 (brain, 67kDa)	Cytoplasm	enzyme
GADD45A	growth arrest and DNA-damage-inducible, alpha	Nucleus	other
GPR126	G protein-coupled receptor 126	Plasma Membrane	G-protein coupled receptor
GPR87	G protein-coupled receptor 87	Plasma Membrane	G-protein coupled receptor
GPX2	glutathione peroxidase 2 (gastrointestinal)	Cytoplasm	enzyme
HBEGF	heparin-binding EGF-like growth factor	Extracellular Space	growth factor
HIC2	hypermethylated in cancer 2	Nucleus	other
HOTAIRM1	HOXA transcript antisense RNA, myeloid-specific 1	Other	other
HOXA1	homeobox A1	Nucleus	transcription regulator
HSPA4L	heat shock 70kDa protein 4-like	Cytoplasm	other
IFFO2	intermediate filament family orphan 2	Other	other
IL28RA	interferon, lambda receptor 1	Plasma Membrane	transmembrane receptor
INPP1	inositol polyphosphate-1-phosphatase	Cytoplasm	phosphatase
ITGA6	integrin, alpha 6	Plasma Membrane	transmembrane receptor
ITGB6	integrin, beta 6	Plasma Membrane	other
JPH1	junctophilin 1	Plasma Membrane	other
KANK1	KN motif and ankyrin repeat domains 1	Nucleus	transcription regulator
KCNQ5	potassium channel, voltage gated KQT-like subfamily Q, member 5	Plasma Membrane	ion channel
KIAA1804	mixed lineage kinase 4	Other	kinase
KRT14	keratin 14, type I	Cytoplasm	other
KRT15	keratin 15, type I	Cytoplasm	other
KRT16	keratin 16, type I	Cytoplasm	other
KRT4	keratin 4, type II	Cytoplasm	other
KRT5	keratin 5, type II	Cytoplasm	other
KRT9	keratin 9, type I	Other	other
KRTAP3-1	keratin associated protein 3-1	Other	other
LIMA1	LIM domain and actin binding 1	Cytoplasm	other
LNX2	ligand of numb-protein X 2	Other	other
LOC100499467			
LOC100506119			
LOC284023	uncharacterized LOC284023	Other	other
LUZP1	leucine zipper protein 1	Nucleus	other
MAST4	microtubule associated serine/threonine kinase family member 4	Other	kinase
MEST	mesoderm specific transcript	Cytoplasm	peptidase
METAP1	methionyl aminopeptidase 1	Cytoplasm	peptidase
MIR205HG	MIR205 host gene (non-protein coding)	Other	other

MPZL2	myelin protein zero-like 2	Plasma Membrane	other
NDE1	nudE neurodevelopment protein 1	Nucleus	other
NEDD9	neural precursor cell expressed, developmentally down-regulated 9	Nucleus	other
NRG1	neuregulin 1	Extracellular Space	growth factor
NRXN3	neurexin 3	Other	transporter
OLFM4	olfactomedin 4	Extracellular Space	other
PCDH7	protocadherin 7	Plasma Membrane	other
PCDH8	protocadherin 8	Plasma Membrane	other
PEG10	paternally expressed 10	Nucleus	other
PER2	period circadian clock 2	Nucleus	transcription regulator
PERP	PERP, TP53 apoptosis effector	Plasma Membrane	other
PITPNM3	PITPNM family member 3	Cytoplasm	transporter
PKP2	plakophilin 2	Plasma Membrane	other
PLCXD2	phosphatidylinositol-specific phospholipase C, X domain containing 2	Other	enzyme
PLEKHA7	pleckstrin homology domain containing, family A member 7	Cytoplasm	other
POLR1D	polymerase (RNA) I polypeptide D, 16kDa	Nucleus	enzyme
PPARGC1A	peroxisome proliferator-activated receptor gamma, coactivator 1 alpha	Nucleus	transcription regulator
PPP1R13L	protein phosphatase 1, regulatory subunit 13 like	Nucleus	transcription regulator
PPP1R14C	protein phosphatase 1, regulatory (inhibitor) subunit 14C	Cytoplasm	other
PROM2	prominin 2	Plasma Membrane	transmembrane receptor
PTPRZ1	protein tyrosine phosphatase, receptor-type, Z polypeptide 1	Plasma Membrane	phosphatase
RAP2B	RAP2B, member of RAS oncogene family	Plasma Membrane	enzyme
RASSF10	Ras association (RalGDS/AF-6) domain family (N-terminal) member 10	Other	other
RNASE7	ribonuclease, RNase A family, 7	Extracellular Space	enzyme
RNF128	ring finger protein 128, E3 ubiquitin protein ligase	Cytoplasm	other
RNF39	ring finger protein 39	Cytoplasm	other
SCUBE2	signal peptide, CUB domain, EGF-like 2	Extracellular Space	other
SDC1	syndecan 1	Plasma Membrane	enzyme
SDC4	syndecan 4	Plasma Membrane	other
SERPINB5	serpin peptidase inhibitor, clade B (ovalbumin), member 5	Extracellular Space	other
SGMS2	sphingomyelin synthase 2	Plasma Membrane	enzyme
SHISA2	shisa family member 2	Other	other
SLC14A1	solute carrier family 14 (urea transporter), member 1 (Kidd blood group)	Plasma Membrane	transporter
SLC2A1	solute carrier family 2 (facilitated glucose transporter), member 1	Plasma Membrane	transporter
SOX2	SRY (sex determining region Y)-box 2	Nucleus	transcription regulator

SPATA18	spermatogenesis associated 18	Cytoplasm	other
ST3GAL5	ST3 beta-galactoside alpha-2,3-sialyltransferase 5	Cytoplasm	enzyme
ST6GALNAC2	ST6 (alpha-N-acetyl-neuraminyl-2,3-beta-galactosyl-1,3)-N-acetylgalactosaminide alpha-2,6-sialyltransferase 2	Cytoplasm	enzyme
STK17A	serine/threonine kinase 17a	Nucleus	kinase
STON2	stonin 2	Cytoplasm	other
TAF1D	TATA box binding protein (TBP)-associated factor, RNA polymerase I, D, 41kDa	Nucleus	other
TFCP2L1	transcription factor CP2-like 1	Nucleus	transcription regulator
THAP9-AS1	THAP9 antisense RNA 1	Other	other
THSD4	thrombospondin, type I, domain containing 4	Cytoplasm	other
TIAM1	T-cell lymphoma invasion and metastasis 1	Cytoplasm	other
TP63	tumor protein p63	Nucleus	transcription regulator
TRIM29	tripartite motif containing 29	Cytoplasm	transcription regulator
TSPAN2	tetraspanin 2	Extracellular Space	other
TUFT1	tuftelin 1	Cytoplasm	other
USP31	ubiquitin specific peptidase 31	Nucleus	peptidase
VCL	vinculin	Plasma Membrane	enzyme
WIF1	WNT inhibitory factor 1	Extracellular Space	other
ZFR2	zinc finger RNA binding protein 2	Other	other
ZNF195	zinc finger protein 195	Nucleus	other
ZNF57	zinc finger protein 57	Other	other
ZNF750	zinc finger protein 750	Nucleus	other
ZNF77	zinc finger protein 77	Nucleus	other
ZNRF3	zinc and ring finger 3	Plasma Membrane	enzyme

Supplementary Table 1C. Fibromuscular stroma metagene

Symbol	Entrez Gene Name	Location	Type(s)
ABCA8	ATP-binding cassette, sub-family A (ABC1), member 8	Plasma Membrane	transporter
ABI3BP	ABI family, member 3 (NESH) binding protein	Extracellular Space	other
ADCY9	adenylate cyclase 9	Plasma Membrane	enzyme
AEBP1	AE binding protein 1	Nucleus	peptidase
AGTR1	angiotensin II receptor, type 1	Plasma Membrane	G-protein coupled receptor
ALDH1A1	aldehyde dehydrogenase 1 family, member A1	Cytoplasm	enzyme
ALDH1A2	aldehyde dehydrogenase 1 family, member A2	Cytoplasm	enzyme
ARL3	ADP-ribosylation factor-like 3	Cytoplasm	enzyme
ASPN	asporin	Extracellular Space	other
BGN	biglycan	Extracellular Space	other
C11orf96	chromosome 11 open reading frame 96	Other	other
C1R	complement component 1, r subcomponent	Extracellular Space	peptidase
C1S	complement component 1, s subcomponent	Extracellular Space	peptidase
C7	complement component 7	Extracellular Space	other
CAMK4	calcium/calmodulin-dependent protein kinase IV	Nucleus	kinase
CCDC80	coiled-coil domain containing 80	Nucleus	other
CCDC92	coiled-coil domain containing 92	Cytoplasm	other
CFD	complement factor D (adipsin)	Extracellular Space	peptidase
CHRDL1	chordin-like 1	Extracellular Space	other
CNTN1	contactin 1	Plasma Membrane	enzyme
COL14A1	collagen, type XIV, alpha 1	Extracellular Space	other
COL1A1	collagen, type I, alpha 1	Extracellular Space	other
COL1A2	collagen, type I, alpha 2	Extracellular Space	other
COL3A1	collagen, type III, alpha 1	Extracellular Space	other
COL5A2	collagen, type V, alpha 2	Extracellular Space	other
COL6A1	collagen, type VI, alpha 1	Extracellular Space	other
COL6A2	collagen, type VI, alpha 2	Extracellular Space	other
COL6A3	collagen, type VI, alpha 3	Extracellular Space	other
COL8A1	collagen, type VIII, alpha 1	Extracellular Space	other
CTSK	cathepsin K	Cytoplasm	peptidase
CXCL12	chemokine (C-X-C motif) ligand 12	Extracellular Space	cytokine
CXCL13	chemokine (C-X-C motif) ligand 13	Extracellular Space	cytokine
CYGB	cytoglobin	Cytoplasm	transporter
DACT1	dishevelled-binding antagonist of beta-catenin 1	Cytoplasm	other
DCN	decorin	Extracellular Space	other
DIO2	deiodinase, iodothyronine, type II	Cytoplasm	enzyme
DPT	dermatopontin	Extracellular Space	other
ECM2	extracellular matrix protein 2, female organ and adipocyte specific	Extracellular Space	other
EDNRA	endothelin receptor type A	Plasma Membrane	transmembrane receptor

ENPEP	glutamyl aminopeptidase (aminopeptidase A)	Plasma Membrane	peptidase
EPHA3	EPH receptor A3	Plasma Membrane	kinase
ESR1	estrogen receptor 1	Nucleus	ligand-dependent nuclear receptor
FAM102B	family with sequence similarity 102, member B	Other	other
FAM150B	family with sequence similarity 150, member B	Other	other
FAP	fibroblast activation protein, alpha	Cytoplasm	peptidase
FBLN1	fibulin 1	Extracellular Space	other
FBLN2	fibulin 2	Extracellular Space	other
FBN1	fibrillin 1	Extracellular Space	other
FUT8	fucosyltransferase 8 (alpha (1,6) fucosyltransferase)	Cytoplasm	enzyme
GEM	GTP binding protein overexpressed in skeletal muscle	Plasma Membrane	enzyme
GNG2	guanine nucleotide binding protein (G protein), gamma 2	Plasma Membrane	enzyme
GPC3	glypican 3	Plasma Membrane	other
GPM6B	glycoprotein M6B	Plasma Membrane	other
GPNMB	glycoprotein (transmembrane) nmb	Plasma Membrane	enzyme
GPR133	G protein-coupled receptor 133	Plasma Membrane	G-protein coupled receptor
GTDC1	glycosyltransferase-like domain containing 1	Other	other
HEPH	hephaestin	Plasma Membrane	transporter
HGF	hepatocyte growth factor (hepapoietin A; scatter factor)	Extracellular Space	growth factor
HS6ST2	heparan sulfate 6-O-sulfotransferase 2	Plasma Membrane	enzyme
HSD11B1	hydroxysteroid (11-beta) dehydrogenase 1	Cytoplasm	enzyme
IGF1	insulin-like growth factor 1 (somatomedin C)	Extracellular Space	growth factor
IGFBP5	insulin-like growth factor binding protein 5	Extracellular Space	other
IGFBP6	insulin-like growth factor binding protein 6	Extracellular Space	other
ISL1	ISL LIM homeobox 1	Nucleus	transcription regulator
JAZF1	JAZF zinc finger 1	Nucleus	transcription regulator
KCNE4	potassium channel, voltage gated subfamily E regulatory beta subunit 4	Plasma Membrane	ion channel
KDELC1	KDEL (Lys-Asp-Glu-Leu) containing 1	Cytoplasm	other
KIRREL	kin of IRRE like (Drosophila)	Plasma Membrane	other
KLF12	Kruppel-like factor 12	Nucleus	transcription regulator
LOC100505971 LOC72839	uncharacterized LOC728392	Other	other
LRFN5	leucine rich repeat and fibronectin type III domain containing 5	Nucleus	other
LTBP1	latent transforming growth factor beta binding protein 1	Extracellular Space	other
LUM	lumican	Extracellular Space	other

MCM6	minichromosome maintenance complex component 6	Nucleus	enzyme
MFAP4	microfibrillar-associated protein 4	Extracellular Space	other
MIR100HG	mir-100-let-7a-2 cluster host gene (non-protein coding)	Other	other
MKX	mohawk homeobox	Nucleus	other
MLLT11	myeloid/lymphoid or mixed-lineage leukemia (trithorax homolog, Drosophila); translocated to, 11	Cytoplasm	other
MMP2	matrix metallopeptidase 2 (gelatinase A, 72kDa gelatinase, 72kDa type IV collagenase)	Extracellular Space	peptidase
MOXD1	monooxygenase, DBH-like 1	Cytoplasm	enzyme
MXRA8	matrix-remodelling associated 8	Other	other
MYH11	myosin, heavy chain 11, smooth muscle	Cytoplasm	other
NDNF	neuron-derived neurotrophic factor	Extracellular Space	other
NID1	nidogen 1	Extracellular Space	other
NR2F1	nuclear receptor subfamily 2, group F, member 1	Nucleus	ligand-dependent nuclear receptor
NRK	Nik related kinase	Other	kinase
OGN	osteoglycin	Extracellular Space	growth factor
OLFML3	olfactomedin-like 3	Extracellular Space	other
PAGE4	P antigen family, member 4 (prostate associated)	Other	other
PAMR1	peptidase domain containing associated with muscle regeneration 1	Extracellular Space	peptidase
PAPLN	papilin, proteoglycan-like sulfated glycoprotein	Extracellular Space	other
PCOLCE	procollagen C-endopeptidase enhancer	Extracellular Space	other
PCOLCE2	procollagen C-endopeptidase enhancer 2	Extracellular Space	other
PDE3A	phosphodiesterase 3A, cGMP-inhibited	Cytoplasm	enzyme
PDGFRA	platelet-derived growth factor receptor, alpha polypeptide	Plasma Membrane	kinase
PENK	proenkephalin	Extracellular Space	other
PGR	progesterone receptor	Nucleus	ligand-dependent nuclear receptor
PLA2G4A	phospholipase A2, group IVA (cytosolic, calcium-dependent)	Cytoplasm	enzyme
PLCL1	phospholipase C-like 1	Cytoplasm	enzyme
PLEKHA2	pleckstrin homology domain containing, family A (phosphoinositide binding specific) member 2	Cytoplasm	other
PRKAR2B	protein kinase, cAMP-dependent, regulatory, type II, beta	Cytoplasm	kinase
PRKG1	protein kinase, cGMP-dependent, type I	Cytoplasm	kinase
PRRX1	paired related homeobox 1	Nucleus	transcription regulator
PRSS35	protease, serine, 35	Extracellular Space	peptidase
PTGDS	prostaglandin D2 synthase 21kDa (brain)	Cytoplasm	enzyme
RBM24	RNA binding motif protein 24	Other	other
RERG	RAS-like, estrogen-regulated, growth inhibitor	Nucleus	enzyme

RNF152	ring finger protein 152	Cytoplasm	enzyme
RPRM	reprimo, TP53 dependent G2 arrest mediator candidate	Cytoplasm	other
RSPO3	R-spondin 3	Extracellular Space	kinase
S100A4	S100 calcium binding protein A4	Cytoplasm	other
S1PR3	sphingosine-1-phosphate receptor 3	Plasma Membrane	G-protein coupled receptor
SCN7A	sodium channel, voltage gated, type VII alpha subunit	Plasma Membrane	ion channel
SEPT11			
SERPINF1	serpin peptidase inhibitor, clade F (alpha-2 antiplasmin, pigment epithelium derived factor), member 1	Extracellular Space	other
SFRP2	secreted frizzled-related protein 2	Plasma Membrane	transmembrane receptor
SGIP1	SH3-domain GRB2-like (endophilin) interacting protein 1	Cytoplasm	other
SLC24A3	solute carrier family 24 (sodium/potassium/calcium exchanger), member 3	Plasma Membrane	transporter
SLIT2	slit homolog 2 (Drosophila)	Extracellular Space	other
SMTNL2	smoothelin-like 2	Other	other
SNX18	sorting nexin 18	Cytoplasm	transporter
SPOCK3	sparc/osteonectin, cwcv and kazal-like domains proteoglycan (testican) 3	Extracellular Space	other
SPON1	spondin 1, extracellular matrix protein	Extracellular Space	other
SRPX2	sushi-repeat containing protein, X-linked 2	Cytoplasm	other
SYNDIG1	synapse differentiation inducing 1	Plasma Membrane	other
SYNM	synemin, intermediate filament protein	Cytoplasm	other
SYT11	synaptotagmin XI	Cytoplasm	transporter
TCEAL7	transcription elongation factor A (SII)-like 7	Nucleus	transcription regulator
THBS2	thrombospondin 2	Extracellular Space	other
TMEM100	transmembrane protein 100	Plasma Membrane	other
TMEM119	transmembrane protein 119	Cytoplasm	other
TMEM200B	transmembrane protein 200B	Other	other
TWIST1	twist family bHLH transcription factor 1	Nucleus	transcription regulator
TWIST2	twist family bHLH transcription factor 2	Nucleus	transcription regulator
WIPF1	WAS/WASL interacting protein family, member 1	Cytoplasm	other
ZCCHC24	zinc finger, CCHC domain containing 24	Other	other
ZEB2	zinc finger E-box binding homeobox 2	Nucleus	transcription regulator

Supplementary Table 1D. Endothelial stroma metagene

Symbol	Entrez Gene Name	Location	Type(s)
ABCB4	ATP-binding cassette, sub-family B (MDR/TAP), member 4	Plasma Membrane	transporter
ACSL5	acyl-CoA synthetase long-chain family member 5	Cytoplasm	enzyme
ADAMTS9	ADAM metallopeptidase with thrombospondin type 1 motif, 9	Extracellular Space	peptidase
ADORA2A	adenosine A2a receptor	Plasma Membrane	G-protein coupled receptor
APOL3	apolipoprotein L, 3	Cytoplasm	transporter
ARAP3	ArfGAP with RhoGAP domain, ankyrin repeat and PH domain 3	Cytoplasm	other
ARHGAP31	Rho GTPase activating protein 31	Cytoplasm	other
ARRB1	arrestin, beta 1	Cytoplasm	other
C3AR1	complement component 3a receptor 1	Plasma Membrane	G-protein coupled receptor
CCDC50	coiled-coil domain containing 50	Cytoplasm	other
CCL22	chemokine (C-C motif) ligand 22	Extracellular Space	cytokine
CCL4	chemokine (C-C motif) ligand 4	Extracellular Space	cytokine
CD80	CD80 molecule	Plasma Membrane	transmembrane receptor
CD83	CD83 molecule	Plasma Membrane	transmembrane receptor
CFP	complement factor properdin	Extracellular Space	other
CX3CL1	chemokine (C-X3-C motif) ligand 1	Extracellular Space	cytokine
CYP1A1	cytochrome P450, family 1, subfamily A, polypeptide 1	Cytoplasm	enzyme
EBI3	Epstein-Barr virus induced 3	Extracellular Space	cytokine
EIF4EBP1	eukaryotic translation initiation factor 4E binding protein 1	Cytoplasm	translation regulator
ENG	endoglin	Plasma Membrane	transmembrane receptor
EREG	epiregulin	Extracellular Space	growth factor
ESAM	endothelial cell adhesion molecule	Plasma Membrane	other
FAM101B	family with sequence similarity 101, member B	Other	other
FAR2	fatty acyl CoA reductase 2	Cytoplasm	enzyme
FCER1G	Fc fragment of IgE, high affinity I, receptor for; gamma polypeptide	Plasma Membrane	transmembrane receptor
FCGR2A	Fc fragment of IgG, low affinity IIa, receptor (CD32)	Plasma Membrane	transmembrane receptor
FCHSD2	FCH and double SH3 domains 2	Other	other
FMNL3	formin-like 3	Cytoplasm	other

GLT25D1	collagen beta(1-O)galactosyltransferase 1	Cytoplasm	other
GNAI2	guanine nucleotide binding protein (G protein), alpha inhibiting activity polypeptide 2	Plasma Membrane	other
GPR4	G protein-coupled receptor 4	Plasma Membrane	G-protein coupled receptor
GPR84	G protein-coupled receptor 84	Plasma Membrane	G-protein coupled receptor
GRB2	growth factor receptor-bound protein 2	Cytoplasm	other
HCK	HCK proto-oncogene, Src family tyrosine kinase	Cytoplasm	kinase
HCLS1	hematopoietic cell-specific Lyn substrate 1	Nucleus	other
HPCAL1	hippocalcin-like 1	Cytoplasm	other
ICAM1	intercellular adhesion molecule 1	Plasma Membrane	transmembrane receptor
IDO1	indoleamine 2,3-dioxygenase 1	Cytoplasm	enzyme
IGHM	immunoglobulin heavy constant mu	Plasma Membrane	transmembrane receptor
IL10	interleukin 10	Extracellular Space	cytokine
IL10RA	interleukin 10 receptor, alpha	Plasma Membrane	transmembrane receptor
IL15RA	interleukin 15 receptor, alpha	Plasma Membrane	transmembrane receptor
IL1B	interleukin 1, beta	Extracellular Space	cytokine
IL23A	interleukin 23, alpha subunit p19	Extracellular Space	cytokine
IL2RA	interleukin 2 receptor, alpha	Plasma Membrane	transmembrane receptor
IL2RG	interleukin 2 receptor, gamma	Plasma Membrane	transmembrane receptor
IL3RA	interleukin 3 receptor, alpha (low affinity)	Plasma Membrane	transmembrane receptor
IL4I1	interleukin 4 induced 1	Cytoplasm	enzyme
IRF4	interferon regulatory factor 4	Nucleus	transcription regulator
ITGA5	integrin, alpha 5 (fibronectin receptor, alpha polypeptide)	Plasma Membrane	transmembrane receptor
KCNN2	potassium channel, calcium activated intermediate/small conductance subfamily N alpha, member 2	Plasma Membrane	ion channel
KIF21B	kinesin family member 21B	Cytoplasm	other
KYNU	kynureninase	Cytoplasm	enzyme
LCP2	lymphocyte cytosolic protein 2 (SH2 domain containing leukocyte protein of 76kDa)	Cytoplasm	other
LILRB2	leukocyte immunoglobulin-like receptor, subfamily B (with TM and ITIM domains), member 2	Plasma Membrane	other
LINC00528	long intergenic non-protein coding RNA 528	Other	other
LOC100287723			
LOC100506098	uncharacterized LOC100506098	Other	other
LOC256021			

LOC374443	C-type lectin domain family 2, member D pseudogene	Other	other
LOC400043	uncharacterized LOC400043	Other	other
LOC550643			
MAP3K11	mitogen-activated protein kinase kinase kinase 11	Cytoplasm	kinase
MAP3K3	mitogen-activated protein kinase kinase kinase 3	Cytoplasm	kinase
MCAM	melanoma cell adhesion molecule	Plasma Membrane	other
MCOLN2	mucolipin 2	Plasma Membrane	ion channel
MGLL	monoglyceride lipase	Plasma Membrane	enzyme
MMP9	matrix metallopeptidase 9 (gelatinase B, 92kDa gelatinase, 92kDa type IV collagenase)	Extracellular Space	peptidase
MNDA	myeloid cell nuclear differentiation antigen	Nucleus	other
MS4A14	membrane-spanning 4-domains, subfamily A, member 14	Other	other
MS4A7	membrane-spanning 4-domains, subfamily A, member 7	Other	other
NAPSB	napsin B aspartic peptidase, pseudogene	Other	other
NCF2	neutrophil cytosolic factor 2	Cytoplasm	enzyme
NLRP3	NLR family, pyrin domain containing 3	Cytoplasm	other
NUMB	numb homolog (Drosophila)	Plasma Membrane	other
OLR1	oxidized low density lipoprotein (lectin-like) receptor 1	Plasma Membrane	transmembrane receptor
OPTN	optineurin	Cytoplasm	other
ORAI1	ORAI calcium release-activated calcium modulator 1	Plasma Membrane	ion channel
P2RY2	purinergic receptor P2Y, G-protein coupled, 2	Plasma Membrane	G-protein coupled receptor
P2RY6	pyrimidinergic receptor P2Y, G-protein coupled, 6	Plasma Membrane	G-protein coupled receptor
PARVB	parvin, beta	Cytoplasm	other
PCDH12	protocadherin 12	Plasma Membrane	other
PDE4DIP	phosphodiesterase 4D interacting protein	Cytoplasm	enzyme
PDGFB	platelet-derived growth factor beta polypeptide	Extracellular Space	growth factor
PIK3AP1	phosphoinositide-3-kinase adaptor protein 1	Cytoplasm	other
PIK3R5	phosphoinositide-3-kinase, regulatory subunit 5	Cytoplasm	kinase
PIM2	Pim-2 proto-oncogene, serine/threonine kinase	Other	kinase
PKIG	protein kinase (cAMP-dependent, catalytic) inhibitor gamma	Other	other
PLAC8	placenta-specific 8	Nucleus	other
PLEK	pleckstrin	Cytoplasm	other

PLEKHO1	pleckstrin homology domain containing, family O member 1	Plasma Membrane	other
PLXNA2	plexin A2	Plasma Membrane	transmembrane receptor
PNPLA6	patatin-like phospholipase domain containing 6	Cytoplasm	other
PPP1R18	protein phosphatase 1, regulatory subunit 18	Other	other
PREX1	phosphatidylinositol-3,4,5-trisphosphate-dependent Rac exchange factor 1	Cytoplasm	other
PRKCH	protein kinase C, eta	Cytoplasm	kinase
PRR5L	proline rich 5 like	Cytoplasm	other
RALGAPA2	Ral GTPase activating protein, alpha subunit 2 (catalytic)	Cytoplasm	other
RAPGEF1	Rap guanine nucleotide exchange factor (GEF) 1	Cytoplasm	other
RAPGEF5	Rap guanine nucleotide exchange factor (GEF) 5	Nucleus	other
RASIP1	Ras interacting protein 1	Cytoplasm	other
RASSF2	Ras association (RalGDS/AF-6) domain family member 2	Nucleus	other
RGS3	regulator of G-protein signaling 3	Nucleus	other
RHBDF2	rhomboid 5 homolog 2 (Drosophila)	Cytoplasm	other
RHOG	ras homolog family member G	Cytoplasm	enzyme
RHOJ	ras homolog family member J	Cytoplasm	enzyme
SELL	selectin L	Plasma Membrane	transmembrane receptor
SH2B3	SH2B adaptor protein 3	Plasma Membrane	other
SH3RF3	SH3 domain containing ring finger 3	Other	other
SH3TC1	SH3 domain and tetratricopeptide repeats 1	Extracellular Space	other
SLC35G2	solute carrier family 35, member G2	Cytoplasm	other
SLC43A3	solute carrier family 43, member 3	Extracellular Space	other
SLC7A7	solute carrier family 7 (amino acid transporter light chain, y+L system), member 7	Plasma Membrane	transporter
SLCO4A1	solute carrier organic anion transporter family, member 4A1	Plasma Membrane	transporter
SOX18	SRY (sex determining region Y)-box 18	Nucleus	transcription regulator
SPRY4	sprouty homolog 4 (Drosophila)	Plasma Membrane	other
SRGAP2	SLIT-ROBO Rho GTPase activating protein 2	Cytoplasm	other
SRGN	serglycin	Cytoplasm	other
STAB1	stabilin 1	Plasma Membrane	transporter
STARD4	StAR-related lipid transfer (START) domain containing 4	Cytoplasm	transporter
STX11	syntaxin 11	Plasma Membrane	transporter
SULF2	sulfatase 2	Plasma Membrane	enzyme

TBC1D1	TBC1 (tre-2/USP6, BUB2, cdc16) domain family, member 1	Nucleus	other
TCHH	trichohyalin	Extracellular Space	other
TFEC	transcription factor EC	Nucleus	transcription regulator
TLL1	tolloid-like 1	Extracellular Space	peptidase
TLR2	toll-like receptor 2	Plasma Membrane	transmembrane receptor
TMEM171	transmembrane protein 171	Other	other
TNFAIP8L1	tumor necrosis factor, alpha-induced protein 8-like 1	Cytoplasm	other
TNFRSF1B	tumor necrosis factor receptor superfamily, member 1B	Plasma Membrane	transmembrane receptor
TNIP1	TNFAIP3 interacting protein 1	Nucleus	other
TNIP3	TNFAIP3 interacting protein 3	Other	other
TRIB3	tribbles pseudokinase 3	Nucleus	kinase
TYROBP	TYRO protein tyrosine kinase binding protein	Plasma Membrane	transmembrane receptor
WARS	tryptophanyl-tRNA synthetase	Cytoplasm	enzyme
ZBTB17	zinc finger and BTB domain containing 17	Nucleus	transcription regulator
ZNF71	zinc finger protein 71	Nucleus	other
ZNF805	zinc finger protein 805	Other	other

Supplementary Table 2 - Gene expression datasets included in the study

Dataset	GEO ID	PMID	Sample	Species	Platform	Datamatrix source	Expression value format
Setlur	GSE8402	18505969	472	Human	Human 6k Transcriptionally Informative Gene Panel for DASL Illumina	GEO	log2 and z-log2
Glinsky	na	15067324	80	Human	Affymetrix U95Av2	Provided by authors	log2 and z-log2
Taylor	GSE21034	20579941	131	Human	Affymetrix Human Exon 1.0 ST Array	GEO	log2 and z-log2
Ehro	GSE46691	23826159	545	Human	Affymetrix Human Exon 1.0 ST Array	Single-Channel Array Normalization (SCAN) from CEL files	log2 and z-log2
TCGA-PRAD	na	na	497	Human	RNAseq (Illumina)	UCSC Xena	log2 (x+1) and z-log2 (x+1)
Grasso	GSE35988	22722839	119	Human	Agilent-014850 Whole Human Genome Microarray 4x44K G4112F (Probe Name version)<br>Agilent-012391 Whole Human Genome Oligo Microarray G4112A (Probe Name version)	cBioportal	log2 and z-log2
Beltran	dbGap phs000909.v.p1	26855148	114	Human	RNAseq (Illumina)	cBioportal / dbGaP	log2 and z-log2
Kumar	GSE77930	26928463	176	Human	Agilent-016162 PEDB Whole Human Genome Microarray 4x44K	cBioportal	log2 and z-log2
Massie	GSE18684	21602788	96	Human	Illumina human-6 v2.0 expression beadchip	GEO	log2 and z-log2
Best	GSE2443	16203770	20	Human	Affymetrix Human Genome U133A Array	GEO	log2 and z-log2
Cai	GSE32269	23426182	55	Human	Affymetrix Human Genome U133A Array	GEO	log2 and z-log2
Aytes	GSE53202	24823640	384	Mouse	Illumina MouseWG-6 v2.0 expression beadchip	GEO	log2 and z-log2

na: non applicable

Supplementary Table 3 - Metagene scores in Taylor dataset

Name	GEO ID	group	fibromE	endotE	basE	lumE
GSM528019	PC3		-2.1388783	0.20108776	0.42335603	-4.5625763
GSM528015	DU145		-2.4213579	-0.6886449	0.17548586	-4.5109305
GSM527895	Primary Site	q1	0.3220827	1.6912556	2.946857	-3.8486907
GSM527866	Primary Site	q1	2.1582792	2.832974	0.8153258	-2.8091226
GSM527958	Primary Site	q1	0.37518972	3.085958	1.394746	-2.1225822
GSM527950	Primary Site	q1	-0.2001206	1.4853171	-0.9138869	-2.0359375
GSM527933	Primary Site	q1	1.2643636	1.4933094	0.9565059	-1.6820835
GSM528016	LNCaP		-2.887572	-2.1785104	-1.4375986	-1.5999486
GSM527985	Primary Site	q1	1.1025304	1.9695457	0.64739496	-1.5742369
GSM527892	Primary Site	q1	1.4259614	0.72741073	1.3584807	-1.4514351
GSM527870	Primary Site	q1	2.866141	1.9663076	0.61748326	-1.3584273
GSM527880	Primary Site	q1	2.0215657	3.0678575	-0.8802797	-1.2117591
GSM527860	Primary Site	q1	0.57095057	0.9773384	2.0934415	-1.2000127
GSM527894	Primary Site	q1	-0.4364706	0.42526045	-0.9488388	-1.198541
GSM527995	Primary Site	q1	-1.5225929	0.14910448	-0.9439555	-1.1333174
GSM527907	Primary Site	q1	0.66702783	0.5865913	1.3493847	-1.0205076
GSM527991	Primary Site	q1	1.6203115	1.391125	1.2090117	-0.9345961
GSM527940	Primary Site	q1	-0.7919573	-0.6550838	-0.0113602	-0.8845427
GSM527948	Primary Site	q1	-0.026868	-0.0230259	-1.099118	-0.8503713
GSM528020	VCaP		-2.7642717	-1.9276557	-0.9228028	-0.8179431
GSM527882	Primary Site	q1	1.3651031	0.9518042	-0.8181826	-0.8173167
GSM527874	Primary Site	q1	0.6054948	0.95542467	-0.6683535	-0.7613151
GSM527896	Primary Site	q1	-0.3258154	0.85558194	-1.5021708	-0.7322459
GSM527979	Primary Site	q1	-0.1502371	0.16650198	0.0502147	-0.6909785
GSM527993	Primary Site	q1	-0.6790034	0.7363999	-1.0939851	-0.6431391
GSM527938	Primary Site	q1	0.23384218	1.3347634	1.479832	-0.6414657
GSM527891	Primary Site	q1	-0.1855611	-0.4351046	1.2925599	-0.5724127
GSM527883	Primary Site	q1	0.27417678	1.3976619	0.186292	-0.4719818
GSM527945	Primary Site	q1	1.2935336	1.1899099	0.38278395	-0.4174627
GSM527904	Primary Site	q1	0.848005	0.5472361	1.173958	-0.4172925
GSM527917	Primary Site	q1	-0.9687452	-0.3565199	-0.8189771	-0.397595
GSM527906	Primary Site	q1	-0.5123994	-0.6465194	0.2938582	-0.3806375
GSM527977	Primary Site	q1	0.12835781	0.06681666	-1.195884	-0.3128247
GSM527955	Primary Site	q1	-0.0799269	0.9491561	-0.8219703	-0.2969527
GSM527990	Primary Site	q1	-0.5105646	-0.1017076	0.3641648	-0.2665896
GSM527936	Primary Site	q1	-1.0507452	0.04985849	-0.7101856	-0.2455532
GSM527905	Primary Site	q1	-0.0638411	-0.255231	0.9254856	-0.1682684
GSM527975	Primary Site	q2q3q4	-0.6939283	-0.1626664	-0.517871	-0.1672225
GSM527965	Primary Site	q2q3q4	-1.1903279	0.7251313	-1.3972957	-0.1129708
GSM527987	Primary Site	q2q3q4	-0.0497894	0.33411112	-1.2982657	-0.0801829
GSM527937	Primary Site	q2q3q4	-1.2454761	-0.9804115	-0.7896421	-0.0576112
GSM527942	Primary Site	q2q3q4	0.03754093	0.16079228	0.06325707	-0.0562457
GSM527885	Primary Site	q2q3q4	1.1168449	1.3381977	-0.3185858	-0.0554525
GSM527858	Primary Site	q2q3q4	1.6048499	0.13331011	1.1376842	-0.0477812
GSM527871	Primary Site	q2q3q4	0.02598175	1.3028673	-0.0032667	-0.0277792
GSM527901	Primary Site	q2q3q4	0.14016525	-0.7931005	1.4620504	-0.0205337
GSM527963	Primary Site	q2q3q4	-0.139305	-0.1393992	-0.7978199	-0.0117912

GSM527861	Primary Site	q2q3q4	0.38854694	-0.0871304	0.40577176	0.00248876
GSM527859	Primary Site	q2q3q4	0.6870121	1.1428647	-0.2325591	0.00283085
GSM527952	Primary Site	q2q3q4	0.03993252	0.40650156	-0.1926492	0.01126346
GSM527922	Primary Site	q2q3q4	0.08410794	-0.0400905	0.5072949	0.01874534
GSM527924	Primary Site	q2q3q4	0.39549384	-0.0193288	0.51809597	0.03634619
GSM527911	Primary Site	q2q3q4	0.15849955	1.4942302	-0.0817011	0.03847424
GSM527890	Primary Site	q2q3q4	-0.1315734	0.707133	0.07920565	0.05432097
GSM527972	Primary Site	q2q3q4	0.33409312	0.5121211	1.4139547	0.06340551
GSM527930	Primary Site	q2q3q4	-1.4215715	-0.492037	-0.0556625	0.07430705
GSM527949	Primary Site	q2q3q4	-0.9662961	-0.0880809	-0.1423581	0.07814229
GSM527961	Primary Site	q2q3q4	-0.4717671	0.05751683	-0.6265183	0.10358343
GSM527932	Primary Site	q2q3q4	-0.2516297	-0.0190743	-0.326609	0.12422837
GSM527994	Primary Site	q2q3q4	-1.9960184	-1.6014899	-1.5330611	0.15333533
GSM527974	Primary Site	q2q3q4	-0.2621215	-0.9263569	-1.6322613	0.16711564
GSM527956	Primary Site	q2q3q4	-0.2254643	0.56275326	0.59670883	0.17028221
GSM527989	Primary Site	q2q3q4	0.16631815	-0.5549227	-1.5205017	0.18793124
GSM527986	Primary Site	q2q3q4	-0.7947342	-0.4831638	-0.0592025	0.1939533
GSM527916	Primary Site	q2q3q4	1.0400254	-0.2512629	-0.1355732	0.20271577
GSM527920	Primary Site	q2q3q4	0.75647914	-0.6301329	1.2654625	0.21221957
GSM527902	Primary Site	q2q3q4	0.13214196	-0.1228823	-0.2245593	0.21379857
GSM527925	Primary Site	q2q3q4	1.1285295	0.08705365	1.3387413	0.21445253
GSM527865	Primary Site	q2q3q4	2.15272	0.7901658	1.5574374	0.21577354
GSM527973	Primary Site	q2q3q4	0.16250671	-0.7579437	0.64251906	0.21648587
GSM527971	Primary Site	q2q3q4	0.87815094	0.6031177	1.3843755	0.21682389
GSM527875	Primary Site	q2q3q4	0.65716314	-0.1767203	0.22138229	0.22919706
GSM527888	Primary Site	q2q3q4	0.6252359	0.29468971	-0.5221128	0.235382
GSM527926	Primary Site	q2q3q4	0.6931572	0.06815004	0.62202823	0.24287777
GSM527935	Primary Site	q2q3q4	-0.061903	0.10228543	0.83953285	0.26247767
GSM527927	Primary Site	q2q3q4	0.43335503	0.9049763	2.146741	0.263437
GSM527943	Primary Site	q2q3q4	-0.3993916	-0.9160142	-1.011536	0.2750958
GSM527887	Primary Site	q2q3q4	1.1004888	1.1712345	-0.3326156	0.28363332
GSM527868	Primary Site	q2q3q4	0.4021016	-0.0516819	0.87335914	0.29956862
GSM527863	Primary Site	q2q3q4	0.8745174	0.91801333	0.12276625	0.33141923
GSM527878	Primary Site	q2q3q4	0.6155109	0.38398412	-0.6243854	0.33264348
GSM527898	Primary Site	q2q3q4	0.5515931	-1.0270257	0.08342566	0.33593455
GSM527951	Primary Site	q2q3q4	0.34323248	-0.3421142	0.22647057	0.34478888
GSM527968	Primary Site	q2q3q4	-1.1849902	-0.3766049	-0.6168923	0.35652483
GSM527903	Primary Site	q2q3q4	1.8359101	0.76821977	-1.5534921	0.35915783
GSM527928	Primary Site	q2q3q4	0.5839596	-0.0965581	1.3205291	0.39518538
GSM527944	Primary Site	q2q3q4	-0.8671902	-1.2611223	0.37514848	0.40070572
GSM527929	Primary Site	q2q3q4	-0.6081666	-0.5104781	0.14816286	0.4250222
GSM527873	Primary Site	q2q3q4	0.92977625	-0.3693202	0.44250998	0.431228
GSM527872	Primary Site	q2q3q4	1.4173347	0.870321	0.41900775	0.4319036
GSM527919	Primary Site	q2q3q4	-0.6172544	0.19784243	0.2648726	0.4371876
GSM527946	Primary Site	q2q3q4	0.23360036	0.8936349	-1.020697	0.4405187
GSM527980	Primary Site	q2q3q4	-1.0458035	0.11186692	-0.9988288	0.4648413
GSM527962	Primary Site	q2q3q4	-0.1833019	0.22213295	1.1502566	0.48515233
GSM527893	Primary Site	q2q3q4	-0.3678293	0.0872704	0.6704487	0.49353597
GSM527992	Primary Site	q2q3q4	-0.0013927	-0.2702973	-0.6004136	0.49578977
GSM527934	Primary Site	q2q3q4	-0.9075308	-0.8817924	-1.0544775	0.50079006

GSM527884	Primary Site	q2q3q4	-0.8749857	0.20352337	-1.082138	0.5084597
GSM527881	Primary Site	q2q3q4	0.68644106	0.23918715	0.30592436	0.5160493
GSM527960	Primary Site	q2q3q4	0.96815467	0.65865713	2.4104795	0.5190616
GSM527913	Primary Site	q2q3q4	-0.275975	0.21183796	1.3612678	0.51922244
GSM527908	Primary Site	q2q3q4	0.49226606	-0.0898561	0.00703429	0.5525447
GSM527921	Primary Site	q2q3q4	-0.0762275	-0.8625388	0.05698251	0.57727593
GSM527876	Primary Site	q2q3q4	-0.6502764	-0.5790228	0.09681825	0.58077073
GSM527879	Primary Site	q2q3q4	1.8285629	1.3187716	-0.3804103	0.60504764
GSM527982	Primary Site	q2q3q4	-0.6331835	-0.8407821	-0.0410822	0.6417818
GSM527923	Primary Site	q2q3q4	1.0605266	-0.2587321	1.4251428	0.6810922
GSM527886	Primary Site	q2q3q4	0.91700214	-0.1421067	0.20581341	0.7242269
GSM527877	Primary Site	q2q3q4	0.02336461	0.23559338	-0.2385642	0.72496206
GSM527939	Primary Site	q2q3q4	-1.5943516	-1.3057059	-0.8314023	0.73199165
GSM527984	Primary Site	q2q3q4	-0.0503282	-0.1526883	0.92070776	0.7402422
GSM527915	Primary Site	q2q3q4	-1.4924483	-0.8345397	-0.1361519	0.7785096
GSM527910	Primary Site	q2q3q4	-0.0416988	-0.6695519	-0.3619677	0.81838626
GSM527864	Primary Site	q2q3q4	1.2099369	0.03055385	0.32003665	0.8188684
GSM527918	Primary Site	q2q3q4	0.17295061	-1.4443974	0.8529532	0.8272353
GSM527966	Primary Site	q2q3q4	-1.5971198	-1.9443308	-1.7036861	0.83103865
GSM527941	Primary Site	q2q3q4	-0.7627987	-0.6442093	0.27429792	0.86668944
GSM527931	Primary Site	q2q3q4	0.7919704	-0.6039891	-0.9318502	0.9046686
GSM527947	Primary Site	q2q3q4	-0.8584642	-1.2137387	-1.0277003	0.9219748
GSM527867	Primary Site	q2q3q4	0.16852032	-0.304742	0.5814202	0.9373524
GSM527988	Primary Site	q2q3q4	0.2928919	-1.849039	-1.9800987	0.95717156
GSM527900	Primary Site	q2q3q4	-1.5711699	-1.8377979	-0.4443396	1.0084102
GSM527970	Primary Site	q2q3q4	-0.8054308	-0.9779459	-1.4763597	1.0493543
GSM527862	Primary Site	q2q3q4	0.09121856	-0.8031119	0.5604118	1.0622423
GSM527957	Primary Site	q2q3q4	-0.5871097	-0.7017359	-0.946382	1.064508
GSM527869	Primary Site	q2q3q4	0.8179507	-0.6960697	1.0908173	1.0786573
GSM527912	Primary Site	q2q3q4	-0.6521286	-0.80945	-1.4603814	1.0807004
GSM527889	Primary Site	q2q3q4	0.292026	-0.2512701	0.4688098	1.1211927
GSM527914	Primary Site	q2q3q4	-1.4654139	-1.679154	0.8318773	1.1278609
GSM527959	Primary Site	q2q3q4	-0.6517681	-1.0032244	0.07429941	1.154692
GSM527897	Primary Site	q2q3q4	0.2519352	-1.4751886	-2.012576	1.185872
GSM527954	Primary Site	q2q3q4	-1.0378857	-0.6099325	-0.9035272	1.190051
GSM527899	Primary Site	q2q3q4	-0.9196162	-1.8173794	0.3993827	1.2211263
GSM527953	Primary Site	q2q3q4	-0.4307004	-1.7418371	-1.9570463	1.2335783
GSM527909	Primary Site	q2q3q4	-1.1047453	-1.6608963	-1.7556723	1.2892718

Supplementary Table 4 - Normalized enrichment scores (NES) in Taylor and TCGA datasets

Luminal Metagene

Geneset Name[1]	mean NES[2]	NES in Taylor dataset	FDR q-val in Taylor dataset	NES in TCGA PRAD dataset	FDR q-val in TCGA PRAD dataset
LIU_PROSTATE_CANCER_UP	3.78	3.98	<0.00001	3.58	<0.00001
HALLMARK_ANDROGEN_RESPONSE	3.66	4.07	<0.00001	3.24	<0.00001
NELSON_RESPONSE_TO_ANDROGEN_UP	3.40	3.76	<0.00001	3.04	<0.00001
CHARAFE_BREAST_CANCER_LUMINAL_VS_BASAL_UP	2.85	3.37	<0.00001	2.33	<0.00001
WANG_RESPONSE_TO_ANDROGEN_UP	2.71	3.02	<0.00001	2.39	<0.00001
WALLACE_PROSTATE_CANCER_UP	2.64	2.83	<0.00001	2.45	<0.00001
DOANE_RESPONSE_TO_ANDROGEN_UP	2.60	2.88	<0.00001	2.33	<0.00001
TOMLINS_PROSTATE_CANCER_UP	2.59	3.10	<0.00001	2.08	0.00051
YEGNASUBRAMANIAN_PROSTATE_CANCER	2.42	2.62	<0.00001	2.22	0.00020
SMID_BREAST_CANCER_LUMINAL_B_UP	1.88	1.81	0.0048	1.95	0.0017
TOMLINS_PROSTATE_CANCER_DN	-1.76	-1.61	0.022	-1.92	0.00045
CHARAFE_BREAST_CANCER_LUMINAL_VS_BASAL_DN	-1.89	-1.76	0.0084	-2.03	0.00011
HUPER_BREAST_BASAL_VS_LUMINAL_UP	-1.93	-2.33	0.000052	-1.54	0.023
SMID_BREAST_CANCER_BASAL_UP	-2.15	-2.36	0.000057	-1.95	0.00029
LIU_PROSTATE_CANCER_DN	-2.55	-2.69	<0.00001	-2.41	<0.00001
SMID_BREAST_CANCER_LUMINAL_B_DN	-2.72	-3.17	<0.00001	-2.27	<0.00001
WALLACE_PROSTATE_CANCER_RACE_UP	-3.37	-3.85	<0.00001	-2.90	<0.00001

[1] Geneset names beong to MSigDB from The Broad Institute

[2] NES: normalized enrichment score. NES>0: genesets correlated with LM scores; NES<0: genesets anticorrelated with LM scores

Basal Metagene

Geneset Name[1]	mean NES[2]	NES in Taylor dataset	FDR q-val in Taylor dataset	NES in TCGA PRAD dataset	FDR q-val in TCGA PRAD dataset
LIU_PROSTATE_CANCER_DN	4.22	5.19	<0.00001	3.24	<0.00001
CHARAFE_BREAST_CANCER_LUMINAL_VS_BASAL_DN	3.71	4.67	<0.00001	2.75	<0.00001
TOMLINS_PROSTATE_CANCER_DN	3.13	3.57	<0.00001	2.69	<0.00001
WALLACE_PROSTATE_CANCER_RACE_UP	3.07	3.61	<0.00001	2.53	<0.00001
DOANE_RESPONSE_TO_ANDROGEN_DN	2.41	3.14	<0.00001	1.69	0.0039
FARMER_BREAST_CANCER_BASAL_VS_LULMINAL	2.10	2.56	<0.00001	1.64	0.0065
OUYANG_PROSTATE_CANCER_PROGRESSION_UP	2.04	2.42	0.000029	1.65	0.0060
CHARAFE_BREAST_CANCER_LUMINAL_VS_BASAL_UP	-1.83	-2.02	0.0018	-1.65	0.027
TOMLINS_PROSTATE_CANCER_UP	-2.17	-1.57	0.036	-2.77	<0.00001
WALLACE_PROSTATE_CANCER_UP	-2.25	-1.95	0.0033	-2.54	<0.00001
LIU_PROSTATE_CANCER_UP	-3.36	-3.36	<0.00001	-3.36	<0.00001

[1] Geneset names beong to MSigDB from The Broad Institute

[2] NES: normalized enrichment score. NES>0: genesets correlated with BM scores; NES<0: genesets anticorrelated with BM scores

Supplementary Table 5

GSEA pathways enriched in low LumE tumors

NAME	SIZE	ES	NES	NOM p-val	FDR q-val	FWER p-val
CELL_CYCLE_PROCESS	180	0.51	2.45	0	0	0
CELL_CYCLE_PHASE	158	0.50	2.38	0	0	0
MITOTIC_CELL_CYCLE	143	0.49	2.28	0	0	0
M_PHASE	105	0.50	2.19	0	8.22E-04	0.003
CELL_CYCLE_GO_0007049	288	0.42	2.12	0	0.002190106	0.01
CHROMOSOME_ORGANIZATION_AND_BIOGENESIS	116	0.46	2.06	0	0.004727753	0.026
CHROMOSOME_SEGREGATION	31	0.59	2.06	0	0.004210623	0.027
INTERPHASE_OF_MITOTIC_CELL_CYCLE	59	0.50	2.01	0	0.00685963	0.05
INTERPHASE	64	0.49	2.00	0	0.006587673	0.054
DNA_PACKAGING	32	0.58	2.00	0	0.00604153	0.054
M_PHASE_OF_MITOTIC_CELL_CYCLE	80	0.48	1.99	0	0.006102488	0.06
MICROTUBULE_CYTOSKELETON_ORGANIZATION_AND_BIOGENESIS	34	0.56	1.98	0	0.00614247	0.066
MITOSIS	77	0.47	1.97	0	0.006262263	0.073
MEIOTIC_CELL_CYCLE	31	0.55	1.90	0	0.012421623	0.15
CYTOKINESIS	17	0.64	1.89	0	0.013320416	0.169
MITOTIC_SISTER_CHROMATID_SEGREGATION	15	0.65	1.88	0	0.014351349	0.19
REGULATION_OF_RNA_METABOLIC_PROCESS	420	0.36	1.87	0	0.014414827	0.202
OMOTER	260	0.37	1.87	0	0.014167817	0.208
TRANSCRIPTION_FROM_RNA_POLYMERASE_II_PROMOTER	413	0.35	1.87	0	0.013530311	0.209
CELL_DIVISION	18	0.63	1.86	0.007246377	0.013841205	0.223
CYTOSKELETON_ORGANIZATION_AND_BIOGENESIS	174	0.39	1.86	0	0.01364611	0.232
NEGATIVE_REGULATION_OF_TRANSCRIPTION	157	0.40	1.85	0	0.014449989	0.253
REGULATION_OF_TRANSCRIPTIONDNA_DEPENDENT	412	0.35	1.84	0	0.015988855	0.289
G1_S_TRANSITION_OF_MITOTIC_CELL_CYCLE	26	0.56	1.84	0.001851852	0.01652401	0.309
NEGATIVE_REGULATION_OF_NUCLEOBASE NUCLEOSIDE NUCLEOTIDE_AND_NUCLEIC_ACID_METABOLIC_PROCESS	177	0.38	1.82	0	0.018933319	0.353
REGULATION_OF_TRANSCRIPTION	494	0.34	1.81	0	0.01999169	0.379
SISTER_CHROMATID_SEGREGATION	16	0.62	1.80	0.003663004	0.021116685	0.404
NEGATIVE_REGULATION_OF_RNA_METABOLIC_PROCESS	111	0.40	1.80	0	0.020440519	0.404
RHO_PROTEIN_SIGNAL_TRANSDUCTION	33	0.52	1.79	0.006837607	0.021746699	0.439

PROTEIN_DNA_COMPLEX_ASSEMBLY	44	0.47	1.78	0	0.02481465	0.495
ESTABLISHMENT_AND_OR_MAINTENANCE_OF_CHROMATIN_ARCHITECTURE	71	0.43	1.77	0.001748252	0.02544292	0.522
NEGATIVE_REGULATION_OF_TRANSCRIPTION_DNA_DEPENDENT	111	0.40	1.77	0	0.026130438	0.543
CHROMATIN_ASSEMBLY	15	0.62	1.76	0.005172414	0.02750737	0.573
POSITIVE_REGULATION_OF_NUCLEOBASE NUCLEOSIDE NUCLEOTIDE_AND_NUCLEIC_ACID_METABOLIC_PROCESS	126	0.38	1.75	0	0.030775841	0.617
POSITIVE_REGULATION_OF_RNA_METABOLIC_PROCESS	101	0.39	1.75	0	0.030184586	0.619
POSITIVE_REGULATION_OF_TRANSCRIPTION	117	0.38	1.73	0	0.033751804	0.662
LOCOMOTORY_BEHAVIOR	55	0.44	1.73	0.006980803	0.03335124	0.667
REGULATION_OF_SMALL_GTPASE_MEDIATED_SIGNAL_TRANSDUCTION	18	0.58	1.72	0.003669725	0.034920514	0.693
ACTIN_CYTOSKELETON_ORGANIZATION_AND_BIOGENESIS	82	0.41	1.71	0	0.036770396	0.716
PROTEIN_AMINO_ACID_PHOSPHORYLATION	235	0.35	1.71	0	0.03848281	0.746
ACTIN_FILAMENT_BASED_PROCESS	92	0.39	1.71	0	0.038327113	0.753
STRIATED_MUSCLE_DEVELOPMENT	31	0.49	1.70	0.010291595	0.039726198	0.775
POSITIVE_REGULATION_OF_TRANSCRIPTION DNA_DEPENDENT	99	0.39	1.70	0.001751314	0.03908226	0.775
CELL_CYCLE_CHECKPOINT_GO_0000075	44	0.45	1.70	0.005235602	0.03920752	0.784
MICROTUBULE_BASED_PROCESS	75	0.41	1.68	0.001769912	0.045397796	0.838
RAS_PROTEIN_SIGNAL_TRANSDUCTION	51	0.43	1.68	0.001757469	0.044700705	0.839
NEGATIVE_REGULATION_OF_TRANSCRIPTION_FROM_RNA_POLYMERASE_II_PROMOTER	68	0.41	1.66	0	0.048358735	0.867
EPIDERMAL_GROWTH_FACTOR_RECEPTOR_SIGNALING_PATHWAY	20	0.54	1.66	0.010771993	0.04765272	0.87
CHROMATIN_ASSEMBLY_OR_DISASSEMBLY	25	0.52	1.66	0.003710575	0.049855288	0.89
DNA_RECOMBINATION	41	0.45	1.65	0.005163511	0.053491488	0.915
MITOTIC_CELL_CYCLE_CHECKPOINT	19	0.54	1.64	0.010362694	0.055609573	0.929
MRNA_PROCESSING_GO_0006397	58	0.41	1.64	0.005395684	0.05513925	0.93
SYNAPTIC_TRANSMISSION	151	0.35	1.63	0	0.057733208	0.939
CELL_MATRIX_ADHESION	25	0.50	1.61	0.010752688	0.069654085	0.968
CELL_SUBSTRATE_ADHESION	25	0.50	1.61	0.015957447	0.06903207	0.969
ORGANELLE_ORGANIZATION_AND_BIOGENESIS	422	0.30	1.60	0	0.07072876	0.975
MEIOSIS_I	16	0.55	1.60	0.03505535	0.07084052	0.978
REGULATION_OF_ANATOMICAL_STRUCTURE_MORPHOGENESIS	16	0.55	1.60	0.0198915	0.06973725	0.978

RESPONSE_TO_DNA_DAMAGE_STIMULUS	155	0.34	1.60	0	0.06907753	0.981
SMALL_GTPASE_MEDIATED_SIGNAL_TRANSDUCTION	72	0.39	1.60	0.008576329	0.0697924	0.984
DNA_REPAIR	121	0.35	1.59	0.003284072	0.07136379	0.988
DNA_METABOLIC_PROCESS	233	0.32	1.59	0	0.07242779	0.99
CYTOSKELETON_DEPENDENT_INTRACELLULAR_TRANSPORT	25	0.48	1.59	0.02688172	0.071878314	0.99
TRANSCRIPTION_INITIATION	33	0.45	1.59	0.020109689	0.07075521	0.99
CHROMATIN_MODIFICATION	50	0.41	1.59	0.013913044	0.06991819	0.99
EMBRYONIC_DEVELOPMENT	49	0.42	1.59	0.009124087	0.06922986	0.99
SYNAPTOGENESIS	17	0.52	1.57	0.028318584	0.07726929	0.992
NEGATIVE_REGULATION_OF_CELLULAR_METABOLIC_PROCESS	214	0.32	1.57	0	0.07629458	0.992
RNA_SPLICING	75	0.37	1.57	0.008143323	0.07553642	0.992
REGULATION_OF_MITOSIS	38	0.44	1.56	0.015410959	0.07770692	0.993
PHOSPHORYLATION	267	0.31	1.56	0	0.08137464	0.994
SENSORY_PERCEPTION_OF_CHEMICAL_STIMULUS	17	0.52	1.56	0.025408348	0.080381475	0.994
NEGATIVE_REGULATION_OF_METABOLIC_PROCESS	217	0.32	1.56	0	0.07995542	0.994
POST_TRANSLATIONAL_PROTEIN_MODIFICATION	414	0.30	1.55	0	0.08302254	0.997
POSITIVE_REGULATION_OF_CELLULAR_METABOLIC_PROCESS	182	0.32	1.55	0	0.08369312	0.997
POSITIVE_REGULATION_OF_METABOLIC_PROCESS	189	0.32	1.55	0	0.08346587	0.997
NEGATIVE_REGULATION_OF_CELL_DIFFERENTIATION	24	0.49	1.54	0.021885522	0.08707439	0.998
RESPONSE_TO_EXTERNAL_STIMULUS	214	0.31	1.53	0	0.09174767	0.998
RESPONSE_TO_ENDOGENOUS_STIMULUS	188	0.32	1.53	0.001683502	0.09242971	0.998
PROTEIN_POLYMERIZATION	18	0.51	1.52	0.047202796	0.09360968	0.999
ESTABLISHMENT_AND_OR_MAINTENANCE_OF_CELL_POLARITY	15	0.53	1.52	0.040219378	0.09268767	0.999
MRNA_METABOLIC_PROCESS	69	0.37	1.52	0.00862069	0.094458975	1
IMMUNE_SYSTEM_DEVELOPMENT	54	0.39	1.52	0.014492754	0.09342874	1
REGULATION_OF_JNK_ACTIVITY	16	0.52	1.52	0.04363002	0.09312029	1
HEMOPOIETIC_OR_LYMPHOID_ORGAN_DEVELOPMENT	51	0.40	1.51	0.019434629	0.09600099	1
CELL_ACTIVATION	43	0.41	1.50	0.035587188	0.10430005	1
G_PROTEIN_SIGNALING_COUPLED_TO_CAMP_NUCLEOTIDE_SECON D_MESSENGER	50	0.39	1.50	0.020072993	0.10367896	1
POSITIVE_REGULATION_OF_TRANSCRIPTION_FROM_RNA_POLYMER ASE_II_PROMOTER	55	0.38	1.50	0.024096385	0.1049771	1
LYMPHOCYTE_DIFFERENTIATION	16	0.51	1.49	0.043252595	0.10752717	1

REGULATION_OF_CELL_CYCLE	163	0.31	1.49	0.003322259	0.10665647	1
HEMOPOIESIS	50	0.39	1.49	0.019366197	0.11025172	1
POTASSIUM_ION_TRANSPORT	48	0.40	1.48	0.026890757	0.111885995	1
G_PROTEIN_SIGNALING_COUPLED_TO_CYCLIC_NUCLEOTIDE_SECOND_MESSENGER	77	0.35	1.48	0.022569444	0.112339064	1
TRANSMISSION_OF_NERVE_IMPULSE	164	0.31	1.48	0.004846527	0.11265764	1
DNA_DEPENDENT_DNA_REPLICATION	48	0.39	1.47	0.018281536	0.11463205	1
CELL_SURFACE_RECEPTOR_LINKED_SIGNAL_TRANSDUCTION_GO_0007166	484	0.28	1.47	0	0.11356439	1
PROTEIN_MODIFICATION_BY_SMALL_PROTEIN_CONJUGATION	43	0.41	1.47	0.048526864	0.11498127	1
DNA_REPLICATION	90	0.34	1.47	0.01675042	0.11481604	1
PATTERN_SPECIFICATION_PROCESS	27	0.43	1.46	0.037701976	0.11655899	1
CELL_CYCLE_ARREST_GO_0007050	48	0.38	1.46	0.033216782	0.115512654	1
LEUKOCYTE_ACTIVATION	37	0.40	1.46	0.036777582	0.1183944	1
REGULATION_OF_CELL_DIFFERENTIATION	46	0.39	1.45	0.03846154	0.12205169	1
CAMP_MEDIATED_SIGNALING	51	0.38	1.45	0.03902439	0.12130407	1
INFLAMMATORY_RESPONSE	83	0.34	1.44	0.021996615	0.1250118	1
BEHAVIOR	106	0.32	1.43	0.02058319	0.1382498	1
CYCLIC_NUCLEOTIDE_MEDIATED_SIGNALING	79	0.33	1.41	0.022727273	0.14615668	1
REGULATION_OF_MAP_KINASE_ACTIVITY	53	0.36	1.41	0.0328152	0.14494516	1
G_PROTEIN_COUPLED_RECEPTOR_PROTEIN_SIGNALING_PATHWAY	261	0.28	1.41	0.003378379	0.14505985	1
SKELETAL_DEVELOPMENT	70	0.34	1.39	0.03938356	0.16022082	1
RESPONSE_TO_WOUNDING	125	0.30	1.39	0.027972028	0.15981217	1
RNA_PROCESSING	153	0.29	1.38	0.029363785	0.16640732	1
REGULATION_OF_CELLULAR_COMPONENT_ORGANIZATION_AND_BIOGENESIS	105	0.31	1.36	0.033444814	0.17635168	1
CELL_CELL_SIGNALING	313	0.27	1.36	0.004702194	0.1761828	1
PROTEIN_KINASE_CASCADE	242	0.27	1.34	0.009819968	0.18924665	1
REGULATION_OF_DEVELOPMENTAL_PROCESS	359	0.25	1.29	0.017133957	0.24855524	1

GSEA pathways enriched in non-low LumE tumors

NAME	SIZE	ES	NES	NOM p-val	FDR q-val	FWER p-val
ORGANIC_ACID_METABOLIC_PROCESS	163	-0.56	-2.78	0	0	0
CARBOXYLIC_ACID_METABOLIC_PROCESS	161	-0.56	-2.73	0	0	0
AMINO_ACID_AND_DERIVATIVE_METABOLIC_PROCESS	91	-0.58	-2.60	0	0	0
AMINO_ACID_METABOLIC_PROCESS	72	-0.59	-2.53	0	0	0
NITROGEN_COMPOUND_METABOLIC_PROCESS	136	-0.52	-2.48	0	0	0
NITROGEN_COMPOUND_CATABOLIC_PROCESS	27	-0.70	-2.40	0	0	0
AMINE_METABOLIC_PROCESS	123	-0.50	-2.37	0	0	0
FATTY_ACID_METABOLIC_PROCESS	55	-0.58	-2.36	0	0	0
GOLGI_VESICLE_TRANSPORT	47	-0.61	-2.36	0	0	0
GLYCOPROTEIN_METABOLIC_PROCESS	81	-0.53	-2.34	0	0	0
GLYCOPROTEIN_BIOSYNTHETIC_PROCESS	65	-0.55	-2.33	0	0	0
COFACTOR_METABOLIC_PROCESS	54	-0.58	-2.32	0	0	0
AMINE_CATABOLIC_PROCESS	25	-0.68	-2.29	0	0	0
AMINO_ACID_CATABOLIC_PROCESS	23	-0.69	-2.27	0	0	0
LIPID_METABOLIC_PROCESS	289	-0.42	-2.26	0	0	0
COENZYME_METABOLIC_PROCESS	38	-0.60	-2.26	0	0	0
MONOCARBOXYLIC_ACID_METABOLIC_PROCESS	78	-0.50	-2.21	0	1.02E-04	0.002
LIPOPROTEIN_METABOLIC_PROCESS	32	-0.61	-2.18	0	2.35E-04	0.005
SECRETORY_PATHWAY	77	-0.50	-2.18	0	2.22E-04	0.005
CELLULAR_LIPID_METABOLIC_PROCESS	230	-0.43	-2.18	0	3.00E-04	0.007
LIPOPROTEIN_BIOSYNTHETIC_PROCESS	26	-0.63	-2.15	0	2.85E-04	0.007
PROTEIN_AMINO_ACID_LIPIDATION	24	-0.65	-2.14	0	2.72E-04	0.007
PROTEIN_AMINO_ACID_N_LINKED_GLYCOSYLATION	26	-0.62	-2.11	0	4.52E-04	0.012
SECRETION_BY_CELL	103	-0.46	-2.09	0	6.46E-04	0.018
MACROMOLECULE_BIOSYNTHETIC_PROCESS	283	-0.40	-2.08	0	6.88E-04	0.02
GLYCEROPHOSPHOLIPID_BIOSYNTHETIC_PROCESS	29	-0.59	-2.05	0	0.001021436	0.031
BIOSYNTHETIC_PROCESS	417	-0.37	-2.05	0	9.84E-04	0.031
VESICLE_MEDIATED_TRANSPORT	167	-0.42	-2.03	0	0.001495556	0.047
RESPONSE_TO_OXIDATIVE_STRESS	40	-0.55	-2.02	0	0.001597385	0.052
AROMATIC_COMPOUND_METABOLIC_PROCESS	23	-0.59	-1.96	0	0.003337759	0.108

ER_TO_GOLGI_VESICLE_MEDIATED_TRANSPORT	18	-0.64	-1.95	0	0.003610343	0.121
CATABOLIC_PROCESS	217	-0.38	-1.94	0	0.003955551	0.136
PHOSPHOINOSITIDE_BIOSYNTHETIC_PROCESS	24	-0.58	-1.94	0	0.003937599	0.139
CELLULAR_CATABOLIC_PROCESS	205	-0.39	-1.93	0	0.004202572	0.153
MITOCHONDRION_ORGANIZATION_AND_BIOGENESIS	45	-0.49	-1.91	0	0.004691078	0.175
FATTY_ACID_OXIDATION	17	-0.64	-1.91	0.002262444	0.004585835	0.176
PROTEIN_FOLDING	57	-0.47	-1.90	0	0.005210707	0.2
CARBOXYLIC_ACID_TRANSPORT	35	-0.51	-1.87	0	0.006818615	0.26
CELLULAR_LIPID_CATABOLIC_PROCESS	33	-0.52	-1.86	0	0.007442321	0.29
COFACTOR_BIOSYNTHETIC_PROCESS	21	-0.57	-1.86	0.002288329	0.007597681	0.302
ALCOHOL_METABOLIC_PROCESS	79	-0.42	-1.84	0	0.008786832	0.349
ORGANIC_ACID_TRANSPORT	36	-0.50	-1.84	0	0.008950543	0.362
MITOCHONDRIAL_TRANSPORT	19	-0.57	-1.83	0.008752735	0.009991718	0.398
AMINO_ACID_TRANSPORT	25	-0.53	-1.82	0.002293578	0.010149874	0.409
LIPID_BIOSYNTHETIC_PROCESS	88	-0.41	-1.82	0	0.009942745	0.409
INTRACELLULAR_TRANSPORT	254	-0.34	-1.81	0	0.010783585	0.447
LIPID_CATABOLIC_PROCESS	36	-0.50	-1.80	0.004357298	0.011256761	0.47
SECRETION	156	-0.36	-1.79	0	0.011945435	0.503
INTRACELLULAR_PROTEIN_TRANSPORT	127	-0.37	-1.77	0	0.015349995	0.596
CELLULAR_BIOSYNTHETIC_PROCESS	283	-0.33	-1.75	0	0.017648168	0.649
GENERATION_OF_PRECURSOR_METABOLITES_AND_EN ERGY	110	-0.38	-1.74	0	0.017691337	0.655
GLYCEROPHOSPHOLIPID_METABOLIC_PROCESS	43	-0.45	-1.74	0.009433962	0.017813265	0.663
PHOSPHOLIPID_BIOSYNTHETIC_PROCESS	38	-0.46	-1.74	0.002188184	0.018159801	0.679
PHOSPHOINOSITIDE_METABOLIC_PROCESS	30	-0.49	-1.73	0.007317073	0.017935926	0.684
NITROGEN_COMPOUND_BIOSYNTHETIC_PROCESS	24	-0.52	-1.73	0.006960557	0.018282471	0.695
HETEROCYCLE_METABOLIC_PROCESS	26	-0.49	-1.72	0.002188184	0.019621668	0.731
ESTABLISHMENT_OF_CELLULAR_LOCALIZATION	316	-0.32	-1.70	0	0.022977587	0.78
TRANSLATION	162	-0.35	-1.69	0	0.024521548	0.813
VITAMIN_METABOLIC_PROCESS	16	-0.57	-1.68	0.013100437	0.0250993	0.827
CELLULAR_PROTEIN_COMPLEX_ASSEMBLY	30	-0.47	-1.68	0.008888889	0.025868716	0.842
HORMONE_METABOLIC_PROCESS	28	-0.47	-1.67	0.009852217	0.027036944	0.861
AMINE_TRANSPORT	36	-0.45	-1.66	0.01091703	0.029453067	0.884

PHOSPHOLIPID_METABOLIC_PROCESS	69	-0.39	-1.65	0.006928407	0.030955125	0.898
LIPID_TRANSPORT	26	-0.48	-1.63	0.019512195	0.03622253	0.934
CELLULAR_LOCALIZATION	331	-0.30	-1.62	0	0.037984826	0.942
PEROXISOME_ORGANIZATION_AND_BIOGENESIS	16	-0.54	-1.62	0.029748283	0.037697844	0.942
ESTABLISHMENT_OF_PROTEIN_LOCALIZATION	165	-0.32	-1.60	0.002421308	0.04246176	0.962
STEROID_METABOLIC_PROCESS	67	-0.37	-1.60	0.012376238	0.042787254	0.963
CARBOHYDRATE_METABOLIC_PROCESS	160	-0.32	-1.59	0	0.044456467	0.972
CELLULAR_RESPONSE_TO_STIMULUS	18	-0.51	-1.59	0.033407573	0.04459365	0.974
TRNA_METABOLIC_PROCESS	19	-0.50	-1.58	0.032397408	0.045618348	0.98
PROTEIN_TRANSPORT	137	-0.34	-1.58	0.002531646	0.04568767	0.981
ELECTRON_TRANSPORT_GO_0006118	47	-0.40	-1.56	0.013636364	0.05112939	0.991
CELL_STRUCTURE_DISASSEMBLY_DURING_APOPTOSIS	16	-0.52	-1.56	0.04	0.05257543	0.992
CELLULAR_CARBOHYDRATE_METABOLIC_PROCESS	112	-0.33	-1.55	0	0.054117084	0.994
APOPTOTIC_NUCLEAR_CHANGES	19	-0.49	-1.54	0.02844639	0.05674547	0.995
STEROID_BIOSYNTHETIC_PROCESS	22	-0.48	-1.53	0.032967035	0.06271838	0.996
AMINO_ACID_DERIVATIVE_METABOLIC_PROCESS	20	-0.48	-1.52	0.04988662	0.06263636	0.997
DNA_CATABOLIC_PROCESS	23	-0.47	-1.52	0.043583535	0.06436132	0.998
MEMBRANE_LIPID_METABOLIC_PROCESS	90	-0.33	-1.45	0.021428572	0.09707418	1
PROTEIN_LOCALIZATION	188	-0.29	-1.45	0.002512563	0.096217595	1
MEMBRANE_LIPID_BIOSYNTHETIC_PROCESS	45	-0.37	-1.43	0.045766592	0.10854854	1
PROTEIN_TARGETING	94	-0.31	-1.38	0.037914693	0.13849244	1
MEMBRANE_ORGANIZATION_AND_BIOGENESIS	106	-0.30	-1.37	0.03217822	0.14425813	1
MACROMOLECULE_LOCALIZATION	208	-0.25	-1.27	0.04043127	0.22762077	1

Supplementary Table 6

Table 6A - Target and MOA of top 30 compounds in VCaP

ID	Name	Description	Target	MOA
BRD-K37194137	III606050	Cytochrome P450 inhibitor		Cytochrome P450 inhibitor
BRD-A88080608	equilin	-	HSD17B1	
BRD-A47144777	dihydro-7-desacetyldeoxygedunin	HSP inhibitor	HSP90AA1	HSP inhibitor
BRD-A83859836	tropisetron	-	HTR3A, GLRA1, GLRA2, GLRB, HTR4	
BRD-K07265709	razoxane	Chelating agent	TOP2A, TOP2B	Chelating agent, Topoisomerase inhibitor
BRD-A90515964	guaifenesin	Expectorant		Expectorant
BRD-K09549677	mibefradil	T-type calcium channel blocker	CACNA1G, CACNA1H, CACNA1C, CACNA1I, ANO1, CACNA1D, CACNA1F, CACNA1S, CACNB1, CACNB2, CACNB3, CACNB4, CATSPER1, CATSPER2, CATSPER3, CATSPER4, CYP3A5, CYP3A7, SCN2A, SCN4A, SCN5A, SCN9A	T-type calcium channel blocker
BRD-K73293050	WZ-3146	EGFR inhibitor	EGFR	EGFR inhibitor
BRD-A73605923	mocimycin	Protein synthesis inhibitor		Protein synthesis inhibitor
BRD-K46317332	proadifen	Nitric oxide synthase inhibitor	NOS1	Nitric oxide synthase inhibitor
BRD-A31801025	formestane	Aromatase inhibitor	CYP19A1	Aromatase inhibitor
BRD-K66093087	FGIN-1-43	Benzodiazepine receptor agonist	TSPO	Benzodiazepine receptor agonist
BRD-K42452249	EO-1428	p38 MAPK inhibitor	MAPK11, MAPK14	p38 MAPK inhibitor
BRD-K82036761	sertraline	Serotonin receptor antagonist	SLC6A4, CYP2C19, SLC6A3	Serotonin receptor antagonist
BRD-K23583188	lavendustin-a	EGFR inhibitor	EGFR, LCK	EGFR inhibitor
BRD-K19277754	paroxetine	-	SLC6A4, CHRM1, CHRM2, CHRM3, CHRM4, CHRM5, HTR2A, SLC6A2	
BRD-K51941867	LM-1685	Cyclooxygenase inhibitor	PTGS2	Cyclooxygenase inhibitor
BRD-K59570838	homoveratrylamine	Dopamine analog		
BRD-K80672993	M2-PK-activator		PKM	
BRD-A64479082	quinidine	Sodium channel blocker	SCN5A, ABCB1, CYP2D6, KCNA5, KCNA7, KCNH1, KCNH2, KCNH5, KCNK1, KCNK6, SLC29A4	Sodium channel blocker
BRD-K40782193	QX-222	Sodium channel blocker		Sodium channel blocker
BRD-K08924299	palonosetron	Serotonin receptor antagonist	HTR3A	Serotonin receptor antagonist
BRD-K83508485	FK-888	Tachykinin antagonist	TACR1, TACR2	Tachykinin antagonist
BRD-K02867583	minaprine	Serotonin reuptake inhibitor	HTR2B, SLC6A4, ACHE, CHRM1, DRD1, DRD2, HTR2A, HTR2C, MAOA	Serotonin reuptake inhibitor
BRD-K70914287	BIBX-1382	EGFR inhibitor	EGFR, ERBB2	EGFR inhibitor, Tyrosine kinase inhibitor
BRD-A53952395	prilocaine	Local anesthetic	SCN5A	Local anesthetic
BRD-K56301217	ABT-737	BCL inhibitor	BCL2, BCL2L1, BCL2L2	BCL inhibitor
BRD-K28452084	BRD-K28452084	-	OPRK1	

Table 6B - Target and MOA of top 30 compounds in PC3

ID	Name	Description	Target	MOA
BRD-K16554956	PTB1	AMPK activator	PTPN1	AMPK activator
BRD-K28178212	GW-441756	-	LRRK2, NTRK1	
BRD-A68009927	daunorubicin	-	TOP2A, TOP2B	
BRD-K56301217	ABT-737	BCL inhibitor	BCL2, BCL2L1, BCL2L2	BCL inhibitor
BRD-K28452084	BRD-K28452084	-	OPRK1	
BRD-A53952395	prilocaine	Local anesthetic	SCN5A	Local anesthetic
BRD-K82036761	sertraline	Serotonin receptor antagonist	SLC6A4, CYP2C19, SLC6A3	Serotonin receptor antagonist
BRD-K77677632	SB-200646	Serotonin receptor antagonist	HTR2B, HTR2C	Serotonin receptor antagonist
BRD-K91696562	orantinib	FGFR inhibitor	PDGFRB, AURKA, AURKB, KDR, EGFR, FGFR1, FGFR2, PDGFRA, TBK1	FGFR inhibitor, VEGFR inhibitor, PDGFR receptor inhibitor
BRD-A33168282	sotalol	Adrenergic receptor antagonist	ADRB1, ADRB2, KCNH2	Adrenergic receptor antagonist
BRD-K20285085	fostamatinib	SYK inhibitor	SYK, FLT3, RET	SYK inhibitor
BRD-K12762134	XAV-939	Tankyrase inhibitor	TNKS, TNKS2	Tankyrase inhibitor
BRD-K00184207	GR-206	Aryl hydrocarbon receptor ligand		Aryl hydrocarbon receptor ligand
BRD-A44090213	indoprofen	Cyclooxygenase inhibitor	PTGS1, PTGS2, CXCR1, CXCR2	Cyclooxygenase inhibitor, Prostanoid receptor antagonist
BRD-A78303415	hyoscyamine	-	CHRM1, CHRM2, CHRM3, CHRM4	
BRD-A96485169	EBPC	Aldose reductase inhibitor	AKR1B1	Aldose reductase inhibitor
BRD-A09161221	nomilin	HSP inhibitor		HSP inhibitor
BRD-K25905511	buddleoflavonoloside	Acetylcholinesterase inhibitor		Acetylcholinesterase inhibitor
BRD-U51951544	ZG-10	JNK inhibitor	MAPK8	JNK inhibitor
BRD-K70914287	BIBX-1382	EGFR inhibitor	EGFR, ERBB2	EGFR inhibitor, Tyrosine kinase inhibitor
BRD-K44779798	miglitol	Glucosidase inhibitor	GAA, MGAM, GANAB, GANC, SLC5A4	Glucosidase inhibitor
BRD-A49906757	scopolamine	Acetylcholine receptor antagonist	CHRM2, CHRM1, CHRM3, CHRM4, CHRM5, SI	Acetylcholine receptor antagonist
BRD-K84421793	BRD-K84421793	-	DRD1	
BRD-K64314806	BRD-K64314806	-		
BRD-A65550283	ginsenoside	Steroid hormone receptor agonist		Steroid hormone receptor agonist
BRD-K63641886	cefuroxime	Bacterial cell wall synthesis inhibitor		Bacterial cell wall synthesis inhibitor
BRD-K89125793	tinidazole	Antiprotozoal		Antiprotozoal
BRD-A80641450	FR-139317	Endothelin receptor antagonist	EDNRA	Endothelin receptor antagonist

Table 6C - Top 10 compounds common in PC3 and VCaP

ID	Name	Description	Target	MOA
BRD-K16554956	PTB1	AMPK activator	PTPN1	AMPK activator
BRD-K28178212	GW-441756	-	LRRK2, NTRK1	
BRD-A68009927	daunorubicin	-	TOP2A, TOP2B	
BRD-K56301217	ABT-737	BCL inhibitor	BCL2, BCL2L1, BCL2L2	BCL inhibitor
BRD-K28452084	BRD-K28452084	-	OPRK1	
BRD-A53952395	prilocaine	Local anesthetic	SCN5A	Local anesthetic
BRD-K82036761	sertraline	Serotonin receptor antagonist	SLC6A4, CYP2C19, SLC6A3	Serotonin receptor antagonist
BRD-K77677632	SB-200646	Serotonin receptor antagonist	HTR2B, HTR2C	Serotonin receptor antagonist
BRD-K91696562	orantinib	FGFR inhibitor	PDGFRB, AURKA, AURKB, KDR, EGFR, FGFR1, FGFR2, PDGFRA, TBK1	FGFR inhibitor, VEGFR inhibitor, PDGFR receptor inhibitor
BRD-A33168282	sotalol	Adrenergic receptor antagonist	ADRB1, ADRB2, KCNH2	Adrenergic receptor antagonist
